# Targeting fatty acid synthase suppresses tumor development in *NF2/CDKN2A*-deficient pleural mesothelioma

**DOI:** 10.1038/s41419-026-08481-y

**Published:** 2026-02-28

**Authors:** Sivasundaram Karnan, Akinobu Ota, Muhammad Nazmul Hasan, Hideki Murakami, Md. Lutfur Rahman, Md Wahiduzzaman, Md Towhid Ahmed Shihan, Nushrat Jahan, Lam Quang Vu, Ichiro Hanamura, Akihito Inoko, Miho Riku, Hideaki Ito, Yoshifumi Kaneko, Yinzhi Lin, Toshinori Hyodo, Hiroyuki Konishi, Shinobu Tsuzuki, Yoshitaka Hosokawa

**Affiliations:** 1 Department of Biochemistry, Aichi Medical University School of Medicine, Nagakute, Aichi Japan; 2 Department of Food and Nutritional Environment, College of Human Life and Environment, Kinjo Gakuin University, Nagoya, Aichi Japan; 3 EuGEF Research Foundation, Chattogram, Bangladesh; 4 Department of Internal Medicine, University of Texas Southwestern Medical Center, Dallas, TX USA; 5 Department of Pathology, Aichi Medical University School of Medicine, Nagakute, Aichi Japan; 6 Department of Biochemistry, Emory University School of Medicine, Atlanta, GA USA; 7 Department of Foundations of Medicine, NYU Grossman Long Island School of Medicine, Mineola, NY USA; 8 Division of Hematology, Department of Internal Medicine, Aichi Medical University School of Medicine, Nagakute, Aichi Japan; 9 Department of Microbiology and Immunology, Aichi Medical University School of Medicine, Nagakute, Japan; 10 Department of Pathology, University of Michigan, Ann Arbor, MI USA

**Keywords:** Mesothelioma, High-throughput screening

## Abstract

Pleural mesothelioma (PM) is an uncommon yet deadly cancer linked to asbestos exposure. The lack of effective early diagnosis and treatment leads to reduced life expectancy among patients with PM. This study aims to identify a novel molecular target inhibitor to develop more effective therapeutics for PM. Our drug screening assay showed that the fatty acid synthase (FASN) inhibitor cerulenin demonstrates strong and selective antiproliferative properties against *NF2/CDKN2A(p16)*-deficient PM cells, surpassing the effects of C75, cisplatin or pemetrexed. FASN protein is frequently detected in *NF2/p16*-deficient PM tumor-derived tissues (15/15, 100%), but rarely in *NF2/p16*-intact PM tumors (8/25, 32%). Notably, cerulenin administration successfully reduced the growth of *NF2/p16*-deficient PM tumors in xenografted mice. Cerulenin inhibits mitochondrial fission by targeting dynamin-related protein 1 (DRP1) in *NF2/p16*-deficient cells. Moreover, the disruption of the FASN gene leads to increased ubiquitination of DRP1. These findings suggest that FASN might play a role in the tumorigenesis of PM cells through the regulation of mitochondrial dynamics. This research offers a novel perspective on the potential development of precision medicine for PM.

## Introduction

Pleural mesothelioma (PM) is a highly aggressive neoplasm originating from pleural mesothelial cells, commonly linked to asbestos exposure after a latency period of 30–40 years [1–3]. Diagnosis of PM typically occurs in advanced stages, contributing to a grim prognosis for patients [4]. PM generally responds poorly to radiation and conventional chemotherapy [5]. Recent advancements in the management of the disease, including diagnosis, staging, biomarkers, and treatment strategies, have provided a greater understanding of PM; however, the mortality rate remains high, partially due to late diagnosis and treatment resistance [6, 7].

Recent studies have revealed that PMs are associated with frequent genetic alterations in the neurofibromatosis 2 (*NF2*), cyclin-dependent kinase inhibitor 2 A (*CDKN2A*, *p16*), and BRCA1-associated protein 1 (*BAP1)* tumor suppressor genes [8–11]. Previously, we have observed high expression levels of *FGFR2*, *CD24*, and *CAMK2D* in *NF2-*knockout (KO), *NF2/p16*-double KO (DKO), and *BAP1*-KO mesothelial cell lines, respectively [12–14], suggesting representation of diagnostic and therapeutic targets for PM. Despite the high frequency of *NF2/p16* loss in PM, no promising drug candidates have been established for this subgroup. To address this gap, we performed an anticancer compound screen and identified fatty acid synthase (FASN) inhibition, particularly by cerulenin, as a potent and selective therapeutic vulnerability.

FASN, a key catalytic enzyme involved in long-chain fatty acid synthesis, is highly expressed in several human cancers, including PM [15–17]. FASN activity is necessary for mitochondrial priming [18] and senescence in cancer cells [19]. FASN amplifies mitochondrial ATP synthesis [20] and promotes mitochondrial fission, key processes in the reprogramming of fatty acid metabolism in colon cancer cells [21]. Mitochondria are highly pleomorphic and considered primary mediators of intracellular energy production [22, 23]. Alterations in mitochondrial dynamics are associated with disease progression and drug resistance in various cancers [24–27]; however, the precise role of FASN in mitochondrial biology in PM cells remains unknown.

Inhibiting FASN activity prevents cancer cell proliferation, migration, invasion, cell cycle, signaling pathway, and energy metabolism in breast and colon cancers, diffuse large B-cell lymphomas, melanomas, retinoblastomas, prostate cancers, and mesothelioma cells [17, 28–35]. Cerulenin, which is available as a naturally occurring or pharmacologically synthesized antibiotic, is an effective apoptosis inducer, with mitochondria being the key player in cerulenin-mediated apoptosis [36]. Accordingly, we investigated whether FASN inhibition influences mitochondrial dynamics, focusing on DRP1-driven fission and OPA1/MFN 1/2-mediated fusion in NF2/p16-deficient mesothelial cells. This occurs by disrupting the mitochondrial membrane and dysregulating the mitochondrial membrane proteins [37]. Stepanova et al. demonstrated that NF2 loss correlates with cerulenin sensitivity in mouse schwannoma [38]. Therefore, our study further investigates the mechanistic effects of cerulenin on NF2/p16-deficient human cells, aiming to elucidate its potential therapeutic role and underlying mechanisms in this disease context.

In this study, we have used cerulenin as a pharmacological tool to inhibit FASN, aiming to determine whether FASN blockade selectively suppresses NF2/p16-deficient PM and to elucidate the associated mitochondrial mechanisms. Additionally, considering that cerulenin exhibits a selective antiproliferative effect on PM cells, its role as a potential molecular-targeted anticancer drug was determined.

## Results

### The FASN inhibitor cerulenin selectively suppresses the proliferation of NF2/p16-deficient PM cells

**Fig. 1:**
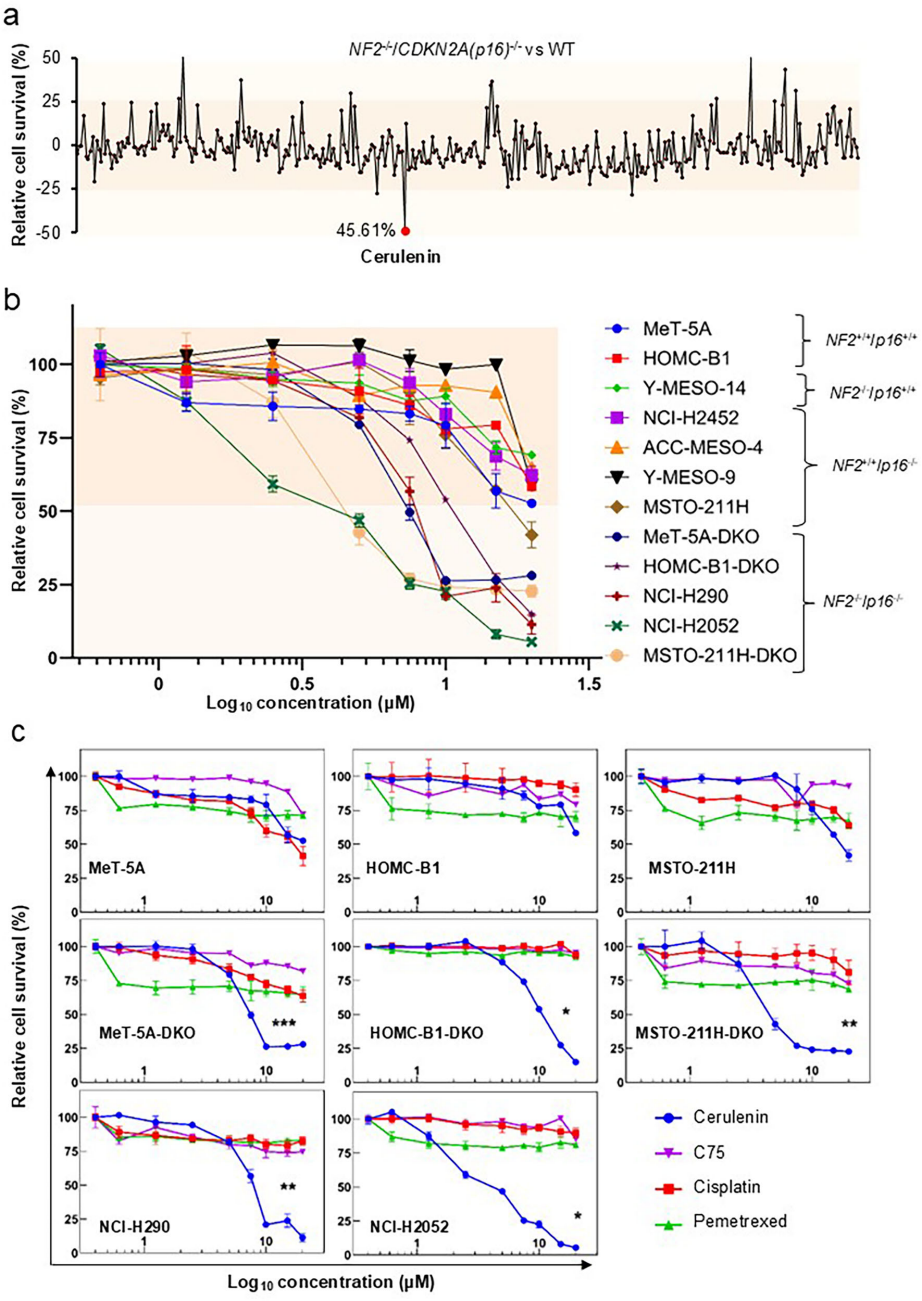
Identification of FASN inhibitor cerulenin in DKO-deficient cells and its effect on PM cell line survival. **a** Screening of compounds from the Screening Committee of Anticancer Drugs (SCADS) library. MeT-5A cells (DKO and NF2/p16-WT [parental] cells) were treated with 364 chemical compounds (10 μM each). Cell survival rates were normalized to the mean optical densities of untreated cells (set as 100%). Results are shown as differences in cell survival percentages between NF2/p16-WT and DKO cells. Red dot indicates>40% reduction in cell viability in DKO cells compared to WT cells. **b** Evaluation of the FASN inhibitor cerulenin on PM cell line survival. Cell survival rates were calculated as described above. **c** Comparison of the effects of cerulenin, C75, cisplatin and pemetrexed on the survival of the indicated PM cell lines after treatment with various concentrations (20, 15, 10, 7.5, 5, 2.5, 1.25, 0.625, and 0 μM) for 72 hours. MTT assays were conducted following the manufacturer’s guidelines, and absorbance was measured at 595 nm using a spectrophotometer. Cell survival percentages were calculated accordingly. Cerulenin, C75, cisplatin, pemetrexed are represented by blue, purple, red and green lines, respectively. Data are presented as mean ± standard error (*n* = 3). Statistical significance was determined by non-linear regression (curve fitting) ^*^p < 0.05, ^**^ *P* < 0.01, ^***^p < 0.001.

Given that we have previously shown differential gene expression between control and *NF2/p16*-double knockout (DKO) isogenic human mesothelial cell clones [13], we attempted to identify an inhibitor that specifically suppresses the proliferation of *NF2/p16*-DKO cells, which are frequently observed in PM. To this end, we performed the MTT assay with the Screening Committee of the Anticancer Drugs library, which comprises 364 chemical compounds (Supplementary Table S1). Our drug screening assay showed that the FASN inhibitor cerulenin, but not C75 (another FASN inhibitor), exhibited the most potent antiproliferative activity against *NF2/p16*-DKO cells (Fig. 1a). To further evaluate cerulenin’s impact on PM cell proliferation, we conducted MTT assays using various mesothelial and PM cell lines. These included *NF2*^+/+^/*p16*^+/+^ (parental MeT-5A and HOMC-B1 cells), *NF2*^−/−^/*p16*^+/+^ (Y-MESO-14), *NF2*^+/+^/*p16*^−/−^ (NCI-2452, ACC-MESO-4, Y-MESO-9, and MSTO-211H), and *NF2*^−/−^/*p16*^−/−^ cells (MeT-5A-DKO, HOMC-B1-DKO, NCI-H290, NCI-H2052, and MSTO-211H-DKO). Interestingly, the average IC_50_ values in the *NF2*/*p16*-DKO mesothelial cell lines and MSTO-211H cells ranged from 5 μM to 7.5 μM, which is lower than that in the *NF2*^+/+^*p16*^+/+^cell lines (~20 μM) (Fig. 1b). This outcome indicates that cerulenin selectively inhibits the proliferation of *NF2*/*p16*-DKO cells. Compared with the antiproliferative effect of cerulenin, treatment with pemetrexed, cisplatin, C75 was relatively ineffective even at higher doses against both the *NF2*^+/+^*p16*^+/+^ and *NF2*^−/−^/*p16*^−/−^ PM cell lines (Fig. 1c). No significant difference in the survival rate between DKO-deficient and DKO WT cells was observed with C75 or desoxyrhaponticin treatment (Supplementary Fig. 1a, b). These findings indicate that cerulenin holds potential as a valuable molecular target for anticancer therapy in PM.

### FASN expression is inversely associated with NF2/p16 expression in human PM tissues

**Fig. 2:**
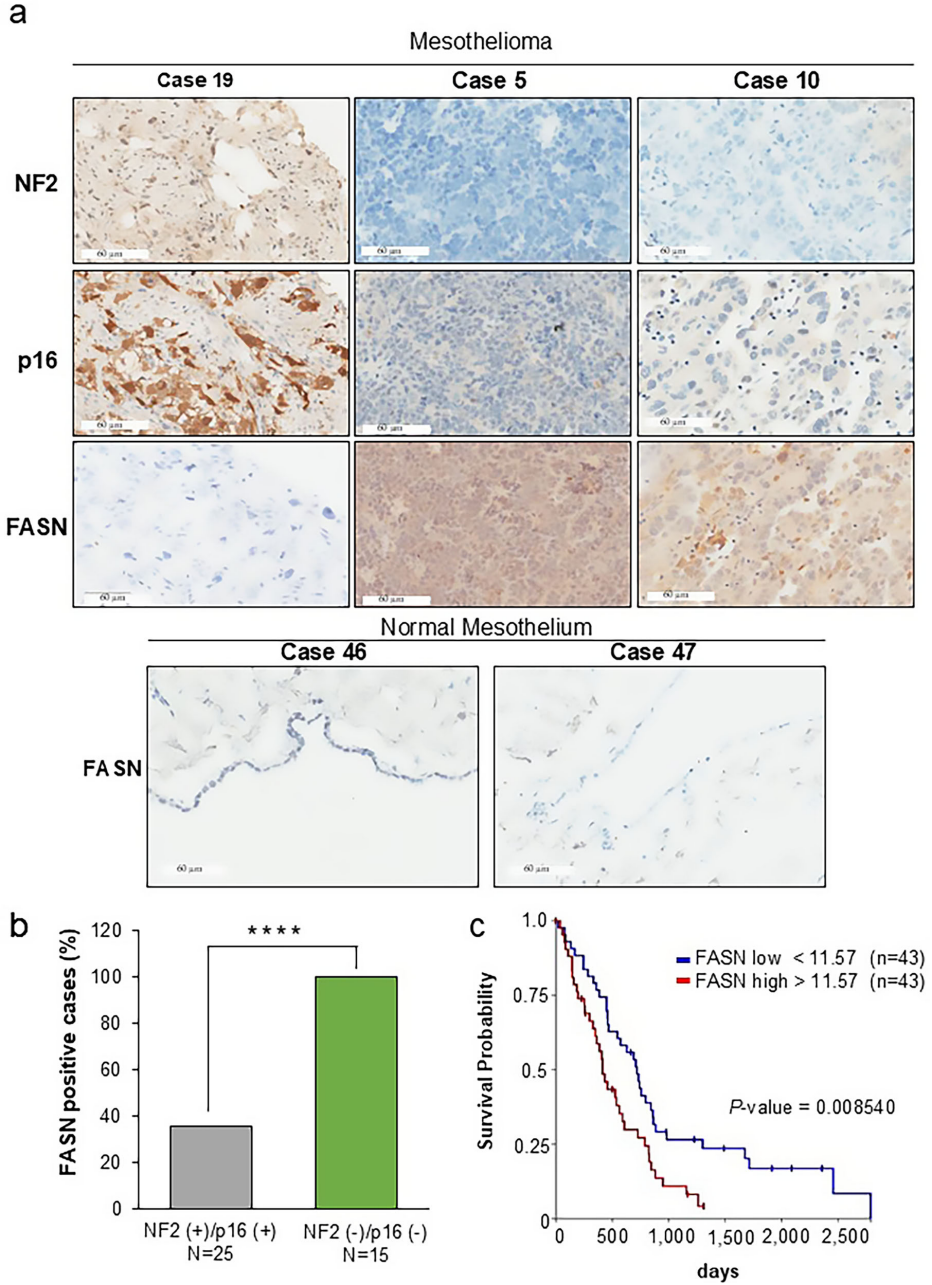
Immunohistochemical (IHC) analysis of FASN expression. **a** Representative IHC findings demonstrating FASN expression in two NF2/P16-negative PM tissues (top panel, cases 5 and 10) in comparison with NF2/P16-positive tissue (top panel, case 19) and two normal mesothelial tissues (bottom panel, cases 46 and 47). **b** Summary of IHC outcomes in PM tissues. The bar graph shows the percentage of total PM cases exhibiting FASN expression by NF2/p16-positive or NF2/p16-negative status. Statistical significance was determined by Fisher’s exact test (two-tailed). ^****^p < 0.0001. **c** Kaplan–Meier analysis was performed to assess the impact of FASN expression on overall survival in TCGA mesothelioma patients, sourced from the UCSC Xena database. TCGA PM patients with high FASN (red) expression (log_2_ > 11.57, *n* = 43) had shorter overall survival than those with low FASN (blue) levels (log_2_ < 11.57, *n* = 43), respectively. (*p* < 0.0085), Scale bar = 60 µm.

Immunohistochemistry was performed using 45 human PM tissue samples and 2 mesothelium samples to assess FASN expression in PM (Supplementary Table S2). IHC analysis identified 15 NF2/P16-negative, 25 NF2/P16-positive, and 5 NF2-positive/P16-negative cases. Microscopic examination revealed 3 strong (3^+^), 14 moderate (2^+^), and 11 weak (1^+^) FASN-positive signals among all cases (Fig. 2a; Supplementary Table S2), whereas no positive signal was observed in the normal mesothelium (Fig. 2a). The frequency of FASN-positive cases was significantly higher in the NF2/p16-negative cases (15/15, 100%) than in the NF2/p16-positive cases (8/25, 32%) (Fig. 2b). Furthermore, FASN expression score was significantly higher in the *NF2/p16*-negative cases (*P* < 0.0024, Supplementary Fig. 2). Notably, our analysis of TCGA PM datasets revealed that patients with higher *FASN* expression exhibited shorter overall survival compared with lower *FASN* expression (Fig. 2c, *P* < 0.008540). These findings strongly indicate a close correlation between elevated FASN expression and poorer prognosis in PM patients.

### Cerulenin suppresses tumor growth of NF/p16-deficient MSTO-211H cells in vivo

**Fig. 3:**
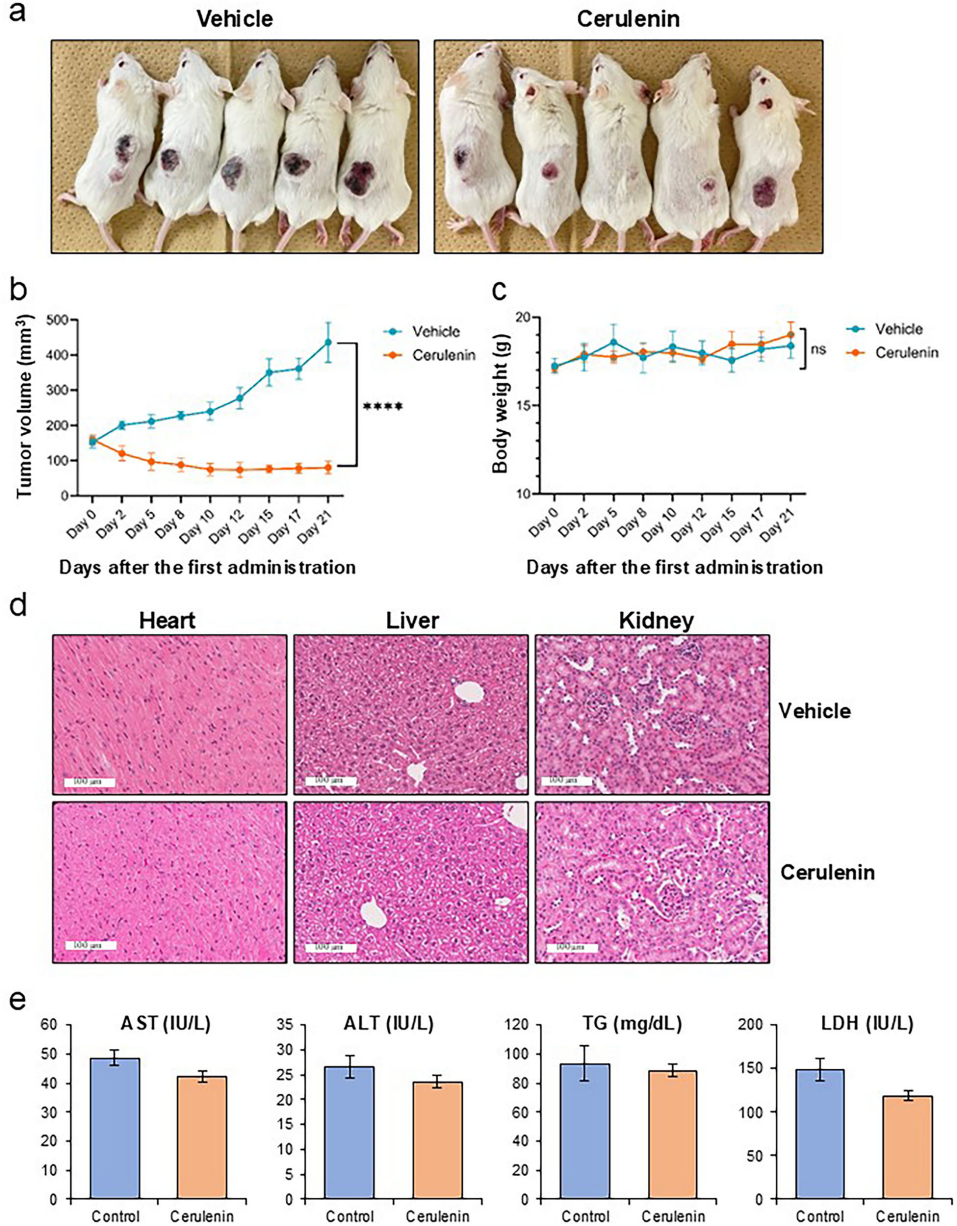
Effect of cerulenin on the growth of MSTO-211H-DKO tumor cells in vivo. The MSTO-211H-DKO cells (5 × 10^6^ cells/mouse) were injected subcutaneously into SCID mice. Following tumor establishment (day 0), cerulenin (20 mg/kg body weight) or PBS vehicle was administered intraperitoneally on days 0, 2, 5, 8, 10, 12, 15, and 17. **a** A representative image depicting tumor-bearing xenografted mice in each group. **b**, **c** Line graphs illustrating (**b**) tumor volume (%) and (**c**) mouse body weight (grams) during cerulenin treatment. Data are presented as mean ± SEM (*n* = 5). ****p = 0.000003, ns = not significant; two-way ANOVA. **d** Representative histochemical images of the heart, liver, and kidney from BALB/c mice after the 14-day observation period (Hematoxylin and Eosin staining, magnification, ×100; scale bar = 100 µm). **e** Alterations in blood chemistry (AST, ALT, TG, and LDH) following cerulenin administration compared with control data; Data are presented as mean ± SE (*n* = 5). *p < 0.05.

The effect on the growth of NF2/p16-deficient MSTO-211H (NF/p16-DKO MSTO-211H) tumors was examined in SCID mice (each *n* = 5 group) to assess the potential of cerulenin for clinical application. Intraperitoneal injection of cerulenin notably inhibited tumor growth in comparison to the vehicle control (Fig. 3a, b). Furthermore, cerulenin treatment did not result in any loss of body weight (Fig. 3c). Subsequent assessment of cerulenin’s safety in normal BALB/c mice (each *n* = 5 group) revealed no noteworthy alterations in body weight (data not presented) or histopathological changes in the heart, liver, or kidneys on day 14 post-treatment (Fig. 3d). Biochemical analyses also showed no alterations in liver function (AST/ALT), kidney function (BUN/Creatinine), electrolytes (Na, K, Cl, Ca, IP) or metabolic, lipid and glucose profile following cerulenin administration (Fig. 3e; Supplementary Table S3). These findings indicate the potential safety and efficacy of cerulenin as a molecular-targeted anticancer agent for managing patients with NF2/p16-deficient PM.

### Cerulenin accelerates the mitochondrial fusion processes and induces apoptosis in NF2/p16-DKO cells

**Fig. 4:**
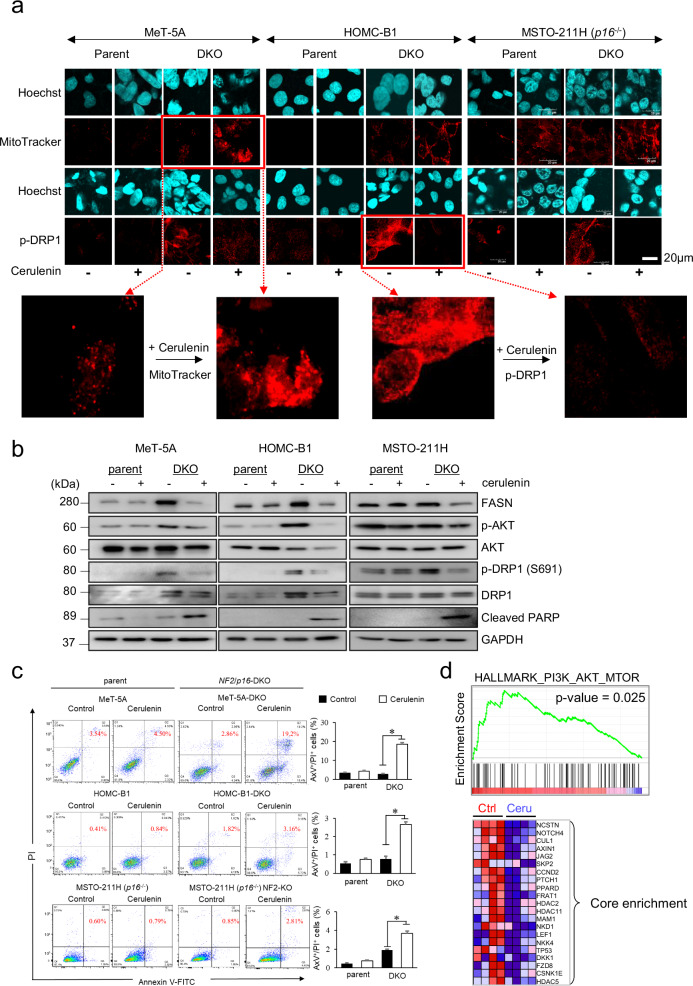
Role of cerulenin in mitochondrial morphology and apoptosis in DKO-deficient PM cells. **a** The mitochondria were visualized using the MitoTracker probe (red). Nuclei were stained with Hoechst (blue), in MeT-5A (upper left panel), HOMC-B1 (upper center panel), and MSTO-211H (upper right panel). Cells were immunostained with Drp1 antibody (red) and Hoechst (blue) in the mitochondria, as depicted in the lower left panels (MeT-5A), lower center panels (HOMC-B1), and lower right panels (MSTO-211H). Two representative magnified view has been illustrated in the bottom part. **b** Protein expression of FASN, p-AKT, AKT, p-Drp1, Drp1, c-PARP, and GAPDH analyzed by Western blotting in MeT-5A, MeT-5A-DKO, HOMC-B1, HOMC-B1-DKO, MSTO-211H, and MSTO-211H-DKO cells treated with cerulenin (7.5 µM) for 48 h. **c** Flow cytometry analysis. Representative results of AxV-PI-based staining are presented on the left. The graphs on the right display the percentage of AxV^+^/PI^+^ apoptotic cells following cerulenin treatment (7.5 µM) for 48 h measured using LSRFortessa X-20 Flow Cytometer (BD Biosciences). Data are mean ± SE (*n* = 3). Asterisks denote significant differences between the DKO-deficient cells (MeT-5A and HOMC-B1 cells and MSTO-211H cells) (*p < 0.05). **d** Gene set enrichment analysis (GSEA). MeT-5A, MeT-5A-DKO, HOMC-B1, and HOMC-B1-DKO cell clones were treated with cerulenin (7.5 µM) or control solvent for 48 h. A cDNA microarray analysis (Agilent) was conducted to obtain a comprehensive gene expression profile. Raw data were analyzed using GSEA with HALLMARK gene sets (C1).

Growing evidence indicates FASN’s role in tumor proliferation through the regulation of energy metabolism. Consequently, we investigated cerulenin’s impact on mitochondrial morphology in *NF2/p16*-DKO cells. We assessed the morphology of the mitochondria using MitoTracker, which permanently binds to mitochondria regardless of cell survival. Confocal microscopy showed that mitochondrial hyperfusion (long tubular network structure) increased after cerulenin treatment in *NF2/p16*-DKO MeT-5A and HOMC-B1 mesothelial cells, whereas no obvious change was observed in the parental cells (Fig. 4a). Similarly, mitochondrial hyperfusion increased after cerulenin treatment in parental MSTO-211H (spontaneously *CDKN2A/p16*-deficient) cells, which was further enhanced in the *NF2/p16*-DKO MSTO-211H cells (Fig. 4a). We examined whether cerulenin influences the phosphorylation status of dynamin-related protein 1 (DRP1), a critical regulator of mitochondrial fission, in *NF2/p16*-DKO cells. Immunofluorescence analysis revealed elevated levels of DRP1 phosphorylation in all *NF2/p16*-DKO cells compared to parental cells; however, cerulenin treatment mitigated these increases in DRP1 phosphorylation levels (Fig. 4a). Western blot analysis demonstrated that cerulenin treatment reduced the phosphorylation levels of both Akt and DRP1, although the extent of reduction varied among the cell lines tested. As expected, FASN protein levels were relatively higher in all *NF2/p16*-DKO cells than in the parental cells tested (Fig. 4b). Kim et al. previously showed TEAD activity and YAP/TAZ downstream target gene *CTGF* and *CYR61* are regulated by FASN expression [39]. Therefore, we sought to examine the effect of FASN in *NF2* and/or *p16*-deficient cells, which exhibit elevated levels of *CTGF* and *CYR61*, reflecting activation of YAP/TEAD signaling (Supplementary Fig. 3). Treatment with cerulenin reduces these levels, suggesting either activation of the Hippo pathway or directly impairing YAP/TEAD function, thus confirming its specificity as an FASN inhibitor. In fact, NF2/p16 loss exhibited high FASN expression levels in the PM cells tested, as compared with NF2- and/or p16-positive PM cells (Supplementary Fig. 4).

To assess cerulenin’s impact on the apoptosis of NF2/p16-double knockout (DKO) cells, we conducted flow cytometry analysis using Annexin V (AxV) and propidium iodide (PI) double staining. The results revealed a significant increase in the Ax^+^/PI^+^ population in NF2/p16-DKO mesothelial and PM cells following cerulenin treatment compared to parental cells (Fig. 4c), suggesting a potential heightened sensitivity of NF2/p16-DKO cells to cerulenin. Similarly, western blot analysis revealed that the cleaved poly (ADP-ribose) polymerase (PARP) levels markedly increased, whereas both FASN protein and phospho-AKT levels decreased after cerulenin treatment in *NF2/p16*-DKO cells (Fig. 4b). To explore the transcriptional changes associated with FASN inhibition, we performed cDNA microarray analysis using parental and *NF2/p16*-DKO MeT-5A and HOMC-B1 cells treated with cerulenin. Gene set enrichment analysis (GSEA) of the resulting expression profiles revealed a significant downregulation of the PI3K-AKT–mTOR signaling pathway in *NF2/p16*-DKO cells following cerulenin treatment (Fig. 4d). These findings strongly indicate that cerulenin selectively triggers apoptosis by targeting oncogenic AKT signaling in *NF2/p16*-DKO cells.

### FASN knockout (TKO) suppresses cell proliferation and DRP1 activity in NF2/p16-DKO MeT-5A cells

**Fig. 5:**
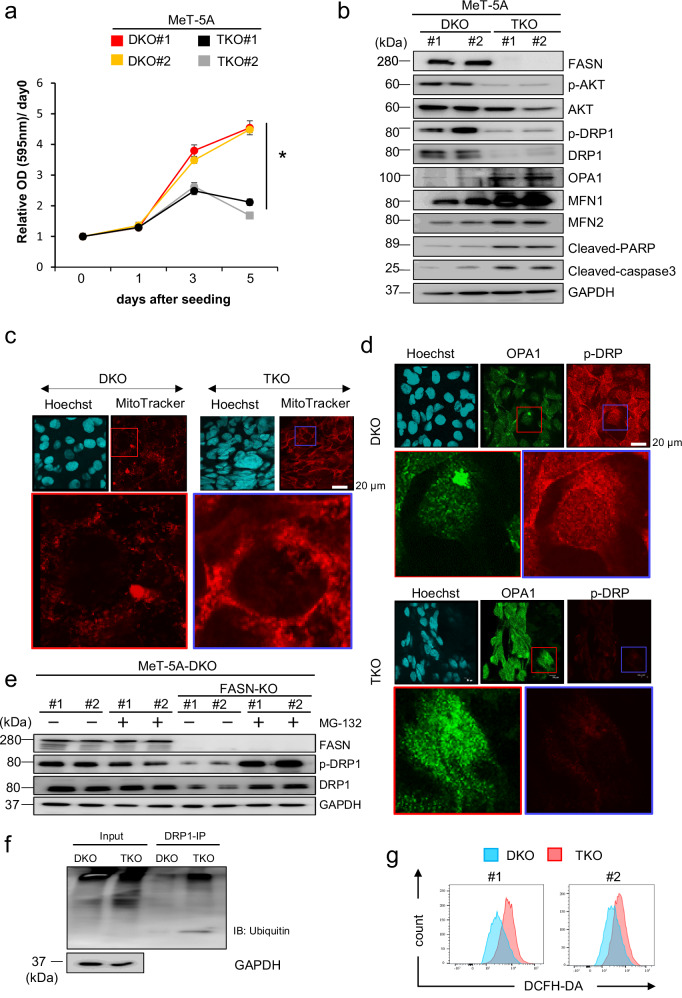
Disruption of *FASN* has adverse effects on mitochondrial morphology and dynamics in PM cells. Cellular phenotype of MeT-5A-DKO/fatty acid synthase (DKO/FASN) triple knockout (TKO) in MeT-5A cells. **a** MTT assay of DKO (#1 and #2) and TKO (#1 and #2) cell clones in MeT-5A cells. The optical density (OD; 595 nm) at each time point (days 0, 1, 3, and 5) is presented as the mean ± SEM (*n* = 6). Growth ratios are expressed relative to the optical densities detected at day 0, arbitrarily defined as 1. **b** Western blot analysis of FASN, p-AKT, AKT, p-Drp1, Drp1, Opa1, MFN1, MFN2, c-PARP, and c-caspase-3 expression in the DKO and TKO cells. **c** Mitochondria labeled with the MitoTracker probe (red; lower panel). Nuclei labeled with Hoechst (blue; upper panel). DKO and TKO are shown in the left and right panels, respectively. **d** Cells immunostained with Opa1 antibody (green) and Hoechst (blue) in the mitochondria, as shown in the left (DKO) and right (TKO) panels. **e** Effects of the proteasome inhibitor MG-132 on FASN, pDrp1, and Drp1 proteins observed in TKO and DKO cells. **f** DKO and TKO cells transfected with ubiquitin expression plasmid followed by MG-132 (10 μM) treatment for 6 hours. Subsequently, each cell lysate was immunoprecipitated with Drp1 and subjected to immunoblot with anti-ubiquitin antibody (P4D1). **g** Effect of DCFH-DH (10 μM, 45 min) on reactive oxygen species production in TKO cells (Red) compared to DKO cells (Blue) in clone #1 (left panel) and #2 (right panel).

*FASN* knockout (hereafter called triple knockout “TKO”) cell clones #1 and #2 were generated using previously established *NF2/p16*-DKO MeT-5A cell clones by targeting exon 3 of the *FASN* gene to further clarify the role of FASN in *NF2/p16*-DKO mesothelial cells (Supplementary Fig. 5a, b). As expected, the FASN protein expression levels were undetected in the TKO #1 and #2 cells (Supplementary Fig. 5c). Further, the effect on the proliferation rate by the disruption of the *FASN* gene was tested. The MTT assay indicated a notably reduced proliferation rate in the TKO cell clones (#1 and #2) compared to the DKO cell clones (Fig. 5a). Furthermore, Western blot analysis revealed a decrease in Akt and DRP1 phosphorylation levels, alongside elevated levels of cleaved PARP and caspase-3 in the TKO cell clones relative to the DKO cell clones (Fig. 5b). Additionally, OPA1, MFN1, and MFN2, which positively regulate mitochondrial fusion, increased at the protein levels in the TKO cell clones (Fig. 5b). Therefore, the effect of *FASN* knockout on the mitochondrial morphology under *NF2/p16*-deficient conditions was examined. Confocal microscopy showed a significant increase in the elongated and tubular structure of the mitochondria in the TKO cells as compared with the DKO cells (Fig. 5c). The protein expression of DRP1 decreased consistently, whereas that of OPA1 increased in the TKO cell clones (Fig. 5d). The effect of proteasome inhibition on DRP1 protein expression was examined to uncover the molecular mechanism underlying the DRP1 expression change in the TKO cells. Western blot analysis demonstrated that both the phosphorylation and protein expression levels of DRP1 were reinstated following treatment with the proteasome inhibitor MG-132 in the TKO cells (Fig. 5e). Furthermore, the immunoprecipitation assay revealed a higher DRP1 ubiquitin level in the TKO cells than in the DKO cells (Fig. 5f). The intracellular reactive oxygen species (ROS), largely generated in the mitochondria, increased in the TKO cells (Fig. 5g), suggesting involvement of FASN expression in the mitochondrial activity, which might affect mitochondrial ROS generation in *NF2/p16*-DKO cells.

## Discussion

Recent studies indicated that *NF2* alterations occur in 40–60% and *CDKN2A* deletions over 90% of PM cases. Combined *NF2* and *CDKN2A* alteration are found in approximately 40–50% of patients, representing a major molecular subset of PM case [40]. In this study, we investigated the involvement of FASN in PM cell proliferation and revealed the selective antiproliferative effect of the FASN inhibitor cerulenin in *NF2*/*p16*-deficient human PM cells. The results revealed that loss of *NF2*/*p16* sensitizes the human mesothelial and PM cells to cerulenin as compared with the *NF2*- and/or *p16*-intact cells. Immunohistochemical analysis also showed detection of FASN expression in *NF2*/*p16*-negative human mesothelioma tissues. The analysis of public data revealed that the overall survival was significantly shorter for patients with PM exhibiting a higher *FASN* expression level. Moreover, cerulenin suppressed the tumor growth of *NF2*/*p16*-deficient human PM cells in vivo without any relevant toxicities.

FASN has been reported as a therapeutic target and an oncogene in several cancers, including breast, pancreatic, and colorectal cancers [15, 41–45]. FASN overexpression is correlated with poor prognosis in HER2-positive ovarian and gastric cancers [46, 47]. FASN overexpression was observed in a subset of PM cell lines and human PM tissues in this study. Additionally, FASN expression was preferentially detected in the *NF2*/*p16*-deficient mesothelial and PM cell lines as compared with the *NF2*/*p16*-intact mesothelial and PM cell lines. Our previous study reported that the loss of *NF2* and *p16* genes significantly enhances anchorage-independent growth and epithelial–mesenchymal transition phenotype in mesothelial cells, in which the cancer-stem cell marker CD24 is upregulated in *NF2*/*p16*-deficient mesothelial and PM cell lines [13, 48] Although the association between *NF2*/*p16* deficiency and FASN overexpression remains unclear, the retardation of the proliferation of *NF2*/*p16*-deficient cells by FASN disruption indicates the possibility of an important role of FASN in *NF2*/*p16*-deficient PM cell survival.

FASN inhibitors differ in their mechanisms of action. Cerulenin irreversibly targets the β-ketoacyl synthase (KS) active site, resulting in potent inhibition and induction of apoptosis. In contrast, C75 is a slow-binding inhibitor with complex pharmacology, blocking multiple FASN domains, including β-KS, enoyl reductase and thioesterase activities. Desoxyrhaponticin inhibits intracellular FASN activity and downregulates FASN gene expression [49, 50]. These differences in affinity and target specificity may explain why cerulenin is more effective than other inhibitors. In our investigation, we observed that the upregulation of FASN expression coincides with the concurrent phosphorylation of oncogenic Akt in *NF2/p16*-DKO cells. Wagner et al. previously reported that the FASN inhibitor impairs receptor-PI3K-mTORC1 signaling in ovarian cancer cells [51]. They also showed that FASN inhibitors impair the phosphatidylinositol 3,4,5-trisphosphate (PIP3) levels, diacylglycerol (DAG), and subsequent PI3K-AKT suppression [51]. We found that cerulenin significantly inactivates the PI3K-Akt-mTOR signaling pathway in *NF2*/*p16*-DKO mesothelial cells. Although it is still unclear whether the membrane lipid composition changes in the *NF2*/*p16*-DKO cells, the loss of *NF2*/*p16* may enhance the proliferation of PM cells via FASN-mediated PI3K-Akt signaling. Indeed, *FASN* knockout in *NF2*/*p16*-DKO cells delays proliferation and decreases Akt phosphorylation as compared with the *FASN*-intact DKO cells. Therefore, it is possible that cerulenin suppresses PI3K-Akt signaling by modulating lipid membrane biogenesis.

Mitochondria are the main organs involved in the generation of cellular fuel ATP through several metabolic processes, including lipid catabolism. Besides, the equilibrium between mitochondrial fission and fusion occurs for the maintenance of mitochondrial functions, which also plays an important role in both normal cells and cancer progression [52–59]. In this current study, the absence of FASN in the DKO (TKO) MeT-5A cells led to an increase in elongated and tubular structured mitochondria, indicative of mitochondrial fusion. Similarly, cerulenin treatment induces mitochondrial fusion formation in *NF2/p16*-deficient (DKO) PM cells.

NF2 is a known upstream regulator of the Hippo pathway, and its disruption reduces Hippo signaling, leading to the accumulation of YAP/TAZ in the nucleus [38]. Together with TEAD, YAP/TAZ promotes the transcription of downstream target genes such as *CTGF* and *CYR61*, thereby enhancing cell proliferation. FASN regulates the palmitoylation and stability of TEAD [39], which sustains the activity of the YAP/TAZ–TEAD complex. In this study, MeT-5A-DKO, HOMC-B1-DKO, *NF2*-deficient MSTO-211H cells exhibit increased expression of YAP target genes, including *CTGF* and *CYR61*, indicating attenuation of Hippo signaling, consistent with previous findings. In contrast, activation of Hippo signaling following cerulenin treatment decreased expression of YAP target genes in *NF2/*p16-deficient PM cells, suggesting that FASN positively regulates YAP activity and its inhibition effectively reduces tumor cell proliferation.

The absence of FASN, as well as FASN inhibition, notably elevated the count of apoptotic cells in DKO PM cells. These findings imply that FASN plays a crucial role in sustaining the survival of PM cells through its involvement in mitochondrial activity. At a molecular level, both the expression and phosphorylation of DRP1 decreased in TKO MeT-5A cells and cerulenin-treated DKO cells. Furthermore, the depletion of FASN in TKO cells resulted in increased protein expression of OPA1, MFN1, and MFN2, all of which promote mitochondrial fusion. Interestingly, proteasome inhibitor MG-132 restored DRP1 protein expression in the TKO cells. Since the ubiquitinated DRP1 level is elevated in TKO cells compared to DKO cells, it suggests a potential role of FASN in stabilizing DRP1 at the protein level.

The present study has some limitations. First, the FASN protein expression was shown to be more frequent in NF2- and/or p16-negative PM tissues as compared to both positive tissues; however, the somatic mutations of *NF2* and/or *p16* genes in the corresponding tissues were not investigated. Therefore, further studies are required to clarify the association between FASN expression and the functional status of *NF2* and/or *p16* in PM. Second, the antitumor effect of cerulenin was demonstrated using only one human PM cell line, MSTO-211H. Hence, additional tests using primary human PM-derived tumors are required for evaluating the efficacy of targeting FASN therapy in PM.

In conclusion, our genome-edited human mesothelial cell model identified that the FASN inhibitor cerulenin preferentially exhibits an antiproliferative effect on PM cells lacking NF2 and/or p16 through regulation of mitochondrial dynamics. These results underscore the potential clinical utility of cerulenin in crafting molecular-targeted therapies for combating PM.

## Materials and methods

### Cell culture

Two immortalized normal human mesothelial cell lines, MeT-5A (pleural mesothelial) and HOMC-B1 (omental mesothelial; epithelioid type), along with eight human mesothelioma cell lines—ACC-MESO-4, Y-MESO-14, Y-MESO-9, NCI-H2452, MSTO-211H, NCI-H290, and NCI-2052—were generously provided by Dr. Y. Sekido from the Division of Molecular Oncology, Aichi Cancer Center Research Institute (Nagoya, Japan). The HOMC-B1 cell lines were maintained according to previously described protocols [13, 60]. The ACC-MESO-4, Y-MESO-14, Y-MESO-9, NCI-H2452, MSTO-211H, NCI-H290, and NCI-2052 cell lines were cultured in RPMI-1640 medium (Wako Pure Chemical Industries, Ltd., Osaka, Japan) supplemented with 10% fetal bovine serum (Sigma) and 1% penicillin-streptomycin (Wako Pure Chemical Industries, Ltd.) at 37°C in a 5% CO2 humidified atmosphere. All tested cell lines are free of mycoplasma contamination.

### Gene KO using the CRISPR/Cas9 system

We employed the CRISPR/Cas9 system to disrupt NF2 expression in the MSTO-211H cell line and FASN expression in the DKO cells, following established procedures [12]. The pSpCas9(BB)-2A-GFP (PX458) plasmid was generously provided by Feng Zhang (plasmid #48138; Addgene, Watertown, MA, USA) [61]. Briefly, sgRNA sequences were chosen using an optimized CRISPR design tool (http://crispr.mit.edu/). The selected sgRNA sequences for NF2 and FASN were 5′-AAACATCTCGTACAGTGACA-3′ in exon 8 and 5′-CCTTCAGCTTGCCGGACCGC-3′ in exon 3, respectively. Plasmids expressing hCas9 and sgRNA were generated by ligating oligonucleotides into the BbsI site of PX458 (NF2/PX458 or FASN/PX458). To establish a knockout (KO) clone, 1 μg of NF2/PX458 plasmid was transfected into the MSTO-211H cell line, and 1 μg of FASN/PX458 plasmid was transfected into MeT-5A-DKO#1 cells (1 × 10^6^ cells) using a 4D-Nucleofector instrument (Lonza Japan, Tokyo, Japan). After 3 days, GFP-expressing cells were sorted using fluorescence-activated cell sorting (BD FACSAria™ III Cell Sorter; BD Biosciences, San Jose, CA, USA). A single clone was selected, expanded, and utilized for the biological assays.

### Screening of anticancer drug library

The Screening Committee of Anticancer Drugs (SCADS) library, consisting of 364 anticancer compounds, was tested on MeT-5A-DKO and parental MeT-5A cells (3000 cells per well) in 96-well plates. The library was kindly provided by the Grant-in-Aid for Scientific Research on Innovative Areas, Scientific Support Programs for Cancer Research, from the Ministry of Education, Culture, Sports, Science and Technology, Japan (http://scads.jfcr.or.jp/kit/index.html). Cells were treated with 10 μM of each compound or DMSO control for 72 hours. Cell viability was assessed using the MTT assay, with absorbance measured at 595 nm and normalized to untreated controls (set at 100%). Compounds that exhibited significant antiproliferative effects on MeT-5A-DKO cells were identified by comparing survival percentages between the two cell types.

### Cell growth assay

The growth rate of the cells was assessed using the MTT assay [62]. Briefly, cells (1 × 10^3^/well) were seeded into 96-well plates and cultured for the specified durations. Subsequently, 10 μL of MTT solution (5 mg/mL; Sigma-Aldrich) was added to each well, and the cells were incubated for 4 hours. Following this, cell lysis buffer was added to dissolve the resultant-colored formazan crystals. The absorbance at 595 nm was measured using a SpectraMAX M5 spectrophotometer (Molecular Devices, Sunnyvale, CA, USA).

### Western blot analysis

Western blotting was conducted following established protocols [13, 63]. The antibodies utilized are detailed in Table S4. Immune complexes were visualized using ImmunoStar LD (Wako Pure Chemical Industries, Ltd.) and imaged with a LAS-4000 image analyzer (GE Healthcare, Tokyo, Japan).

### Annexin V assay

Cells were plated into six-well culture plates (5 × 10^5^ cells/well) and treated with cerulenin (7.5 µg/mL) for 48 hours. Subsequently, the cells were exposed to annexin V (Ax)-FITC and propidium iodide (PI) (10 μg/mL) at 25 °C for 15 minutes. The fluorescence intensities were quantified using fluorescence-activated cell sorting (FACS) analysis (LSRFortessa X-20 Flow Cytometer, BD Biosciences, Franklin Lakes, NJ, USA).

### cDNA microarray analysis

The cDNA microarray analysis was performed following the manufacturer’s protocol (Agilent Technologies), as previously described [14]. Briefly, cDNA synthesis and cRNA labeling were conducted using cyanine 3 (Cy3) dye with the Agilent Low Input Quick Amp Labeling Kit (Agilent Technologies). The Cy3-labeled cRNA was then purified, fragmented, and hybridized onto a Human Gene Expression 8x60K v2 Microarray Chip, which contains 62,969 Entrez Gene RNAs, utilizing the Agilent Gene Expression Hybridization Kit. Raw and normalized microarray data were submitted to the Gene Expression Omnibus database at the National Center for Biotechnology Information (accession number GSE307912; https://www.ncbi.nlm.nih.gov/geo/query/acc.cgi?acc=GSE307912). Gene set enrichment analysis was performed according to the instructions.

### Immunofluorescence

The cells were cultured on glass coverslips and fixed with a 4% paraformaldehyde solution for 20 minutes at 25 °C. Subsequently, the cells were permeabilized with phosphate-buffered saline (PBS) containing 0.1% Triton X-100, blocked in PBS containing 7% serum for 30 minutes, and then incubated with primary antibodies followed by Alexa Fluor-conjugated secondary antibodies (Invitrogen). Cell staining was conducted using MitoTracker (stock solution 1 mM; diluted at 1:10,000) for 1 hour at 37 °C to visualize the mitochondria. Following staining, the cells were washed with PBS and fixed with cold paraformaldehyde (3.2% in PBS) for 20 minutes at room temperature. After additional washing steps, the samples were mounted using PermaFluor, and images were captured using the FLUOVIEW FV3000 Series of Confocal Laser Scanning Microscopes.

### Immunohistochemistry

Immunohistochemical analysis was conducted following previously established protocols [64]. A human mesothelioma tissue array (MS-1001a; US Biomax, Rockville, MD, USA) was utilized. The tissue sections were incubated with primary antibodies (NF2, p16, and FASN antibody, 2 μg/mL). Negative controls were established using normal rabbit immunoglobulin G or by omitting the primary antibody. Immunoreactivity was independently assessed by two investigators (S.K. and H.M.), and staining intensity was graded as strong (+ 3), moderate (+ 2), weak (+ 1), or negative (0).

### Xenograft experiments

All animal experiments were conducted in accordance with the protocols (approval number-2022-20) approved by the ethical committee of Aichi Medical University and followed established guidelines. Female Fox Chase SCID mice (CB17/Icr-Prkdcscid/IcrIcoCrl) were procured from CLEA Japan Inc. (Tokyo, Japan) and housed at the Institute of Animal Experiments in Aichi Medical University under pathogen-free conditions. The mice, aged 6–8 weeks and weighing 17–18 g each, were utilized for the study. Subcutaneous injection of (5 × 10^6^ cells/mouse) was injected subcutaneously into SCID mice. Following tumor establishment (day 0), cerulenin (20 mg/kg body weight) or PBS vehicle was administered intraperitoneally on days 0, 2, 5, 8, 10, 12, 15, and 17. Upon reaching a tumor volume of ~100 mm^3^, the mice were randomly assigned to two groups (treatment and control). Cerulenin (20 mg/kg, administered three times per week) was intraperitoneally administered to mice in the treatment group, while PBS served as the vehicle control in the control group. Tumor measurements were taken every 3–4 days, and tumor volume was calculated using the modified ellipsoid formula (1/2 × length × width^2^). Data are expressed as the mean ± SEM (*n* = 5).

## In vivo effects of cerulenin on BALB/c mice

Eight-week-old female BALB/c mice (*n* = 5) received intraperitoneal injections of cerulenin (20 mg/kg) or PBS on days 0, 3, 5, 7, 10, and 12. Body weights were monitored thrice weekly. Two weeks after the first dose, blood was collected for chemistry analysis, and organs (heart, liver, kidneys) were harvested, fixed, embedded in paraffin, sectioned, and stained with H&E for histological examination.

### DCFH–DA-based DCF assay

Cells were plated into six-well culture plates at a density of 3 × 10^5^ cells per well. The following day, they were exposed to 10 μM DCFH–DA (2’,7’-dichlorodihydrofluorescein diacetate) in the medium for 45 minutes in the absence of light. Subsequently, the DCFH–DA solution was removed, and the cells were washed with PBS. After detachment using trypsin, the cells were suspended in media, centrifuged at 1000 rpm for 5 minutes, and resuspended in PBS in 0.5-mL tubes, which were then placed on ice. The samples were analyzed via FACS using LSRFortessa X-20 Flow Cytometer (BD Biosciences).

### Statistical analysis

The data are presented as mean ± SE (M) values. Statistical significance among groups was assessed using one-way analysis of variance followed by Dunnett’s comparison. All statistical analyses were conducted using GraphPad Prism and/or SPSS 23.0 software (SPSS Inc., Chicago, Illinois, USA).

## Supplementary information

Supplementary Figure

Uncropped Western blots file

Supplementary Table S1

Supplementary Table S2

Supplementary Table S3

Supplementary Table S4

aj-checklist

## Data Availability

All accessible data are provided either in the main manuscript or in the supplementary materials. Complete and unaltered Western blot data utilised in the study are available in the supplementary information. Furthermore, specific inquiries, data, and materials can be obtained upon reasonable request to the corresponding author.
