# Supplementary material for: Targeting fatty acid synthase suppresses tumor development in NF2/CDKN2A-deficient pleural mesothelioma
Source: Cell Death Dis. 2026 Feb 28;17(1):287. doi: 10.1038/s41419-026-08481-y (PMC13031323; doi:10.1038/s41419-026-08481-y)
Supplement: Supplementary file 1 — Supplementary Figure [file 41419_2026_8481_MOESM1_ESM.pdf]

Supplementary Figure

Manuscript Title:
Targeting fatty acid synthase suppresses tumor development in *NF2/CDKN2A*-deficient pleural mesothelioma

Supplementary information includes 5 supplementary figures

a

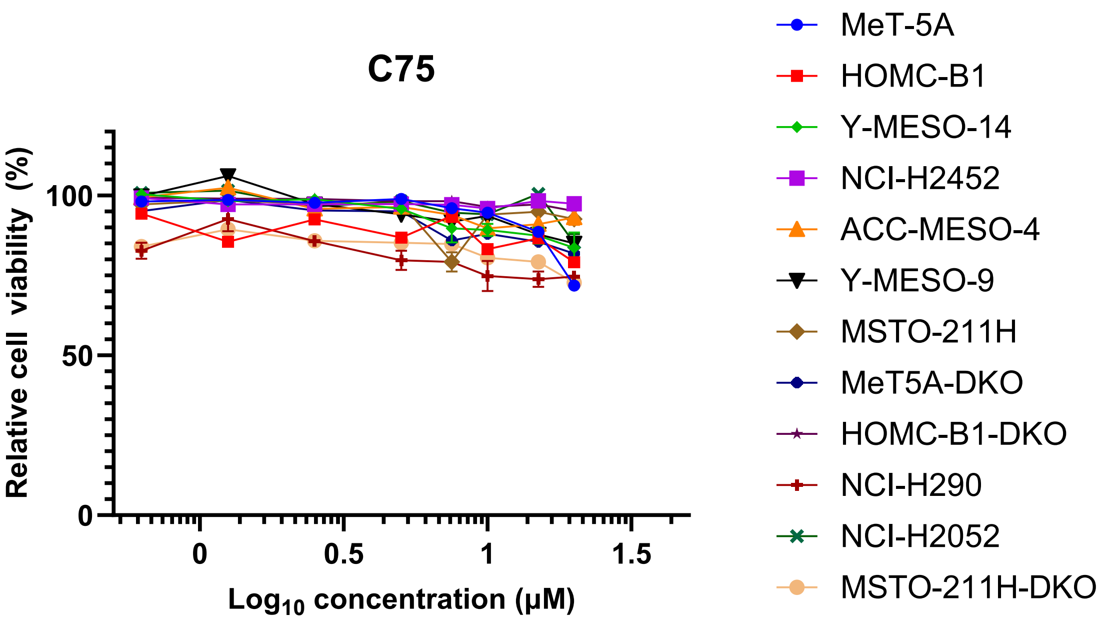

b

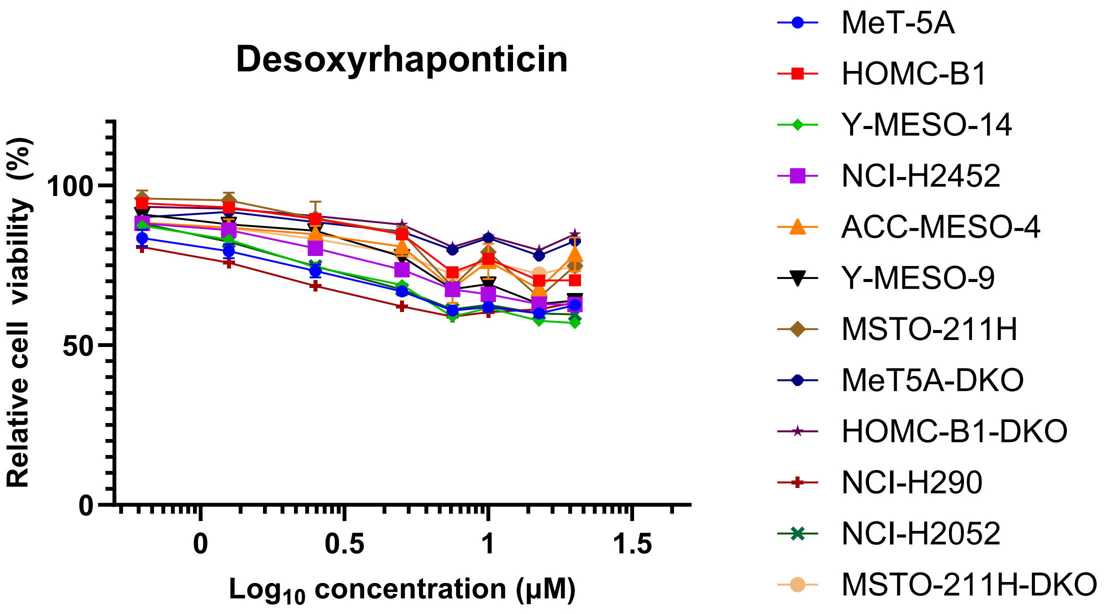

Supplementary Fig. 1. Effect of (a) C75 and (b) Desoxyrhaponticin treatment on cell viability. MeT-5A, HOMC-B1, Y-MESO-14, NCI-H2452, ACC-MESO-4,Y-MESO-9,MSTO-211H MeT-5A-DKO, HOMC-B1-DKO, NCI-290,NCI-2052 and MSTO-211H-DKO cells were seeded in a 96-well plate (cell density, 3×10^3 cells/well). The following day, cells were treated C75 and Desoxyrhaponticin at concentration of 20, 15, 10, 7.5, 5, 2.5, 1.25, 0.625, and 0 μM for 72h. MTT assays were performed according to the manufacturer's instructions. Cell survival percentages were calculated as described above, and absorbance was measured at 595 nm using a spectrophotometer (normalized to 100%). Data are presented as mean ± SE (n = 3). Dose response curves were generated through GraphPad prism and statistical analysis was performed by non-linear regression (curve fitting).

Supplementary Fig. 2

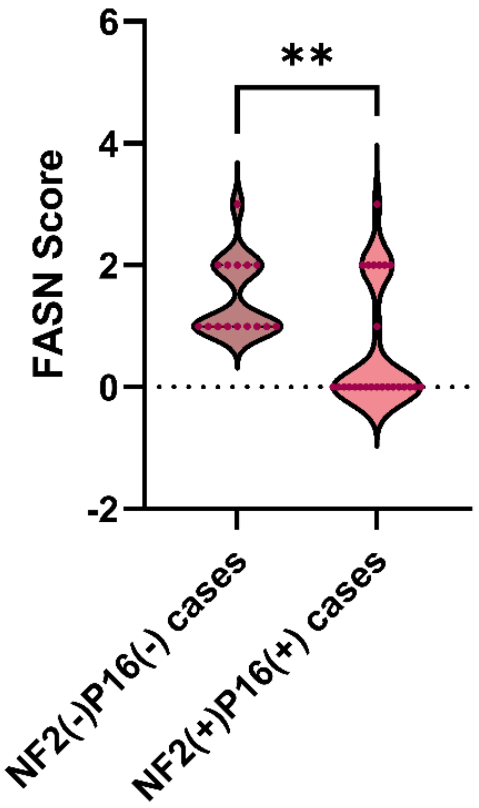

Supplementary Fig. 2. Violin plot showing FASN scores (Y-axis) in NF2/p16-positive and NF2/p16-negative PM tissue samples. Scores were independently evaluated by two investigators. Statistical analysis was performed using the Mann–Whitney test; ***p* < 0.0024.

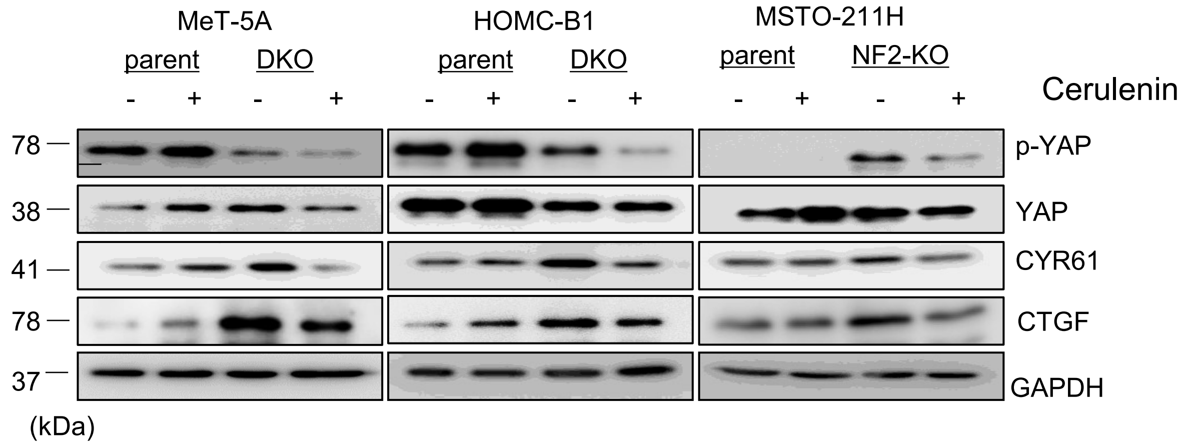

Supplementary Fig. 3. Role of cerulenin in Hippo pathway DKO-deficient PM cells. Protein expression of YAP, p-YAP, CTGF, CYR61, and GAPDH analyzed by Western blotting in MeT-5A, MeT-5A-DKO, HOMC-B1, HOMC-B1-DKO, MSTO-211H, and MSTO-211H-DKO cells treated with cerulenin (7.5 μM) for 48 hour.

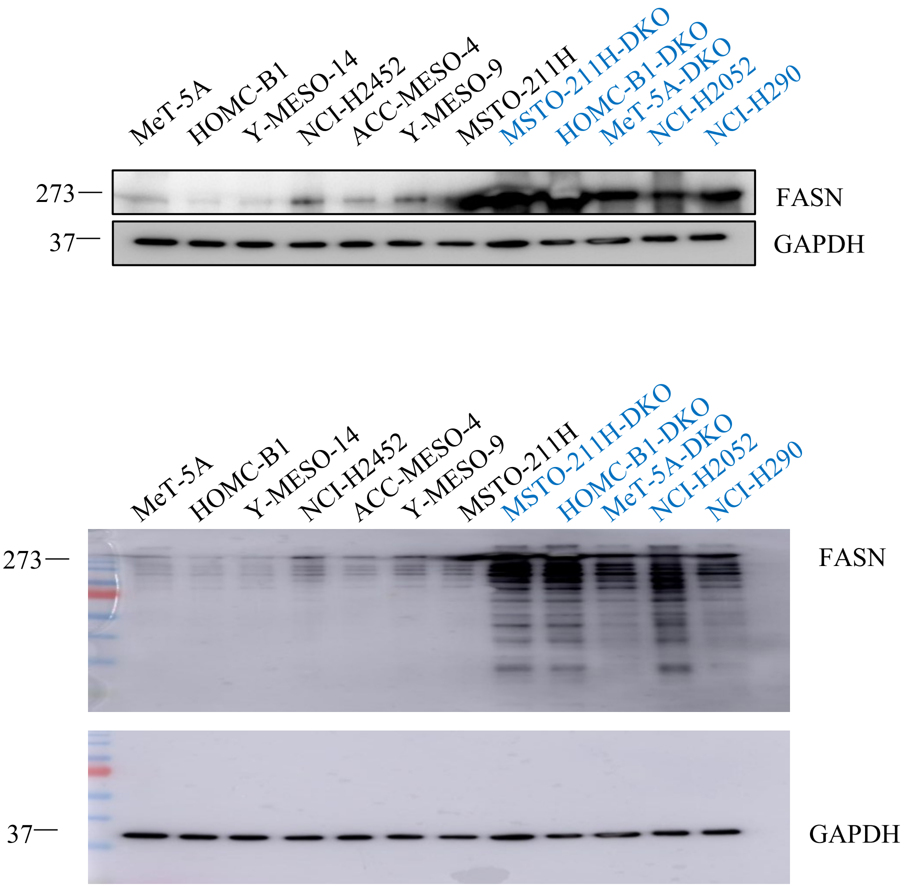

Supplementary Fig. 4. Western blot analysis of FASN expression in the mesothelial and PM cell lines, using GAPDH as the internal control. Blue color indicates NF2/p16-deficient PM cells

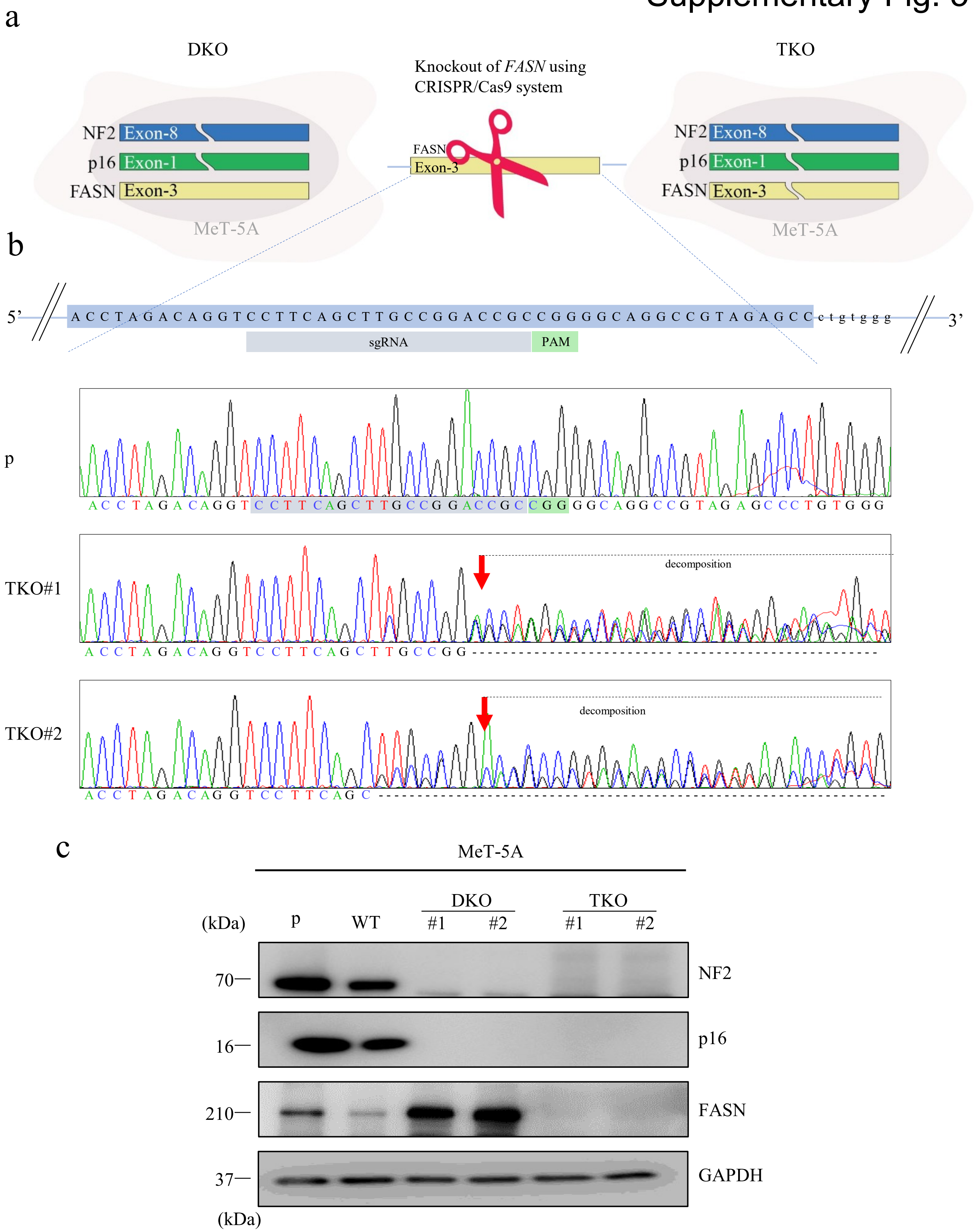

Supplementary Fig. 5. Generation of FASN knockout MeT 5A DKO cells (TKO) using the CRISPR/Cas9 system (a) A specific guide RNA sequence was developed targeting exon 3 of the FASN gene. **(b)** Genomic sequence comparison between TKO#1, TKO#2, and parental cells was performed to analyze alterations in the FASN locus. **(c)** Western blot analysis was conducted to assess the protein expressions of NF2, p16, and FASN.
