# Supplementary material for: Targeting fatty acid synthase suppresses tumor development in NF2/CDKN2A-deficient pleural mesothelioma
Source: Cell Death Dis. 2026 Feb 28;17(1):287. doi: 10.1038/s41419-026-08481-y (PMC13031323; doi:10.1038/s41419-026-08481-y)
Supplement: Supplementary file 2 — Uncropped Western blots file [file 41419_2026_8481_MOESM2_ESM.pdf]

# Uncropped western blots file

**Manuscript Title:**
Targeting fatty acid synthase suppresses tumor development in *NF2/CDKN2A*-deficient pleural mesothelioma

•All the full and uncropped version of the western blot images used in the manuscript has been included in this file (Fig. 4b, Supplementary Fig. 3, Fig. 5b, Fig. 5e and supplementary Fig.5c)

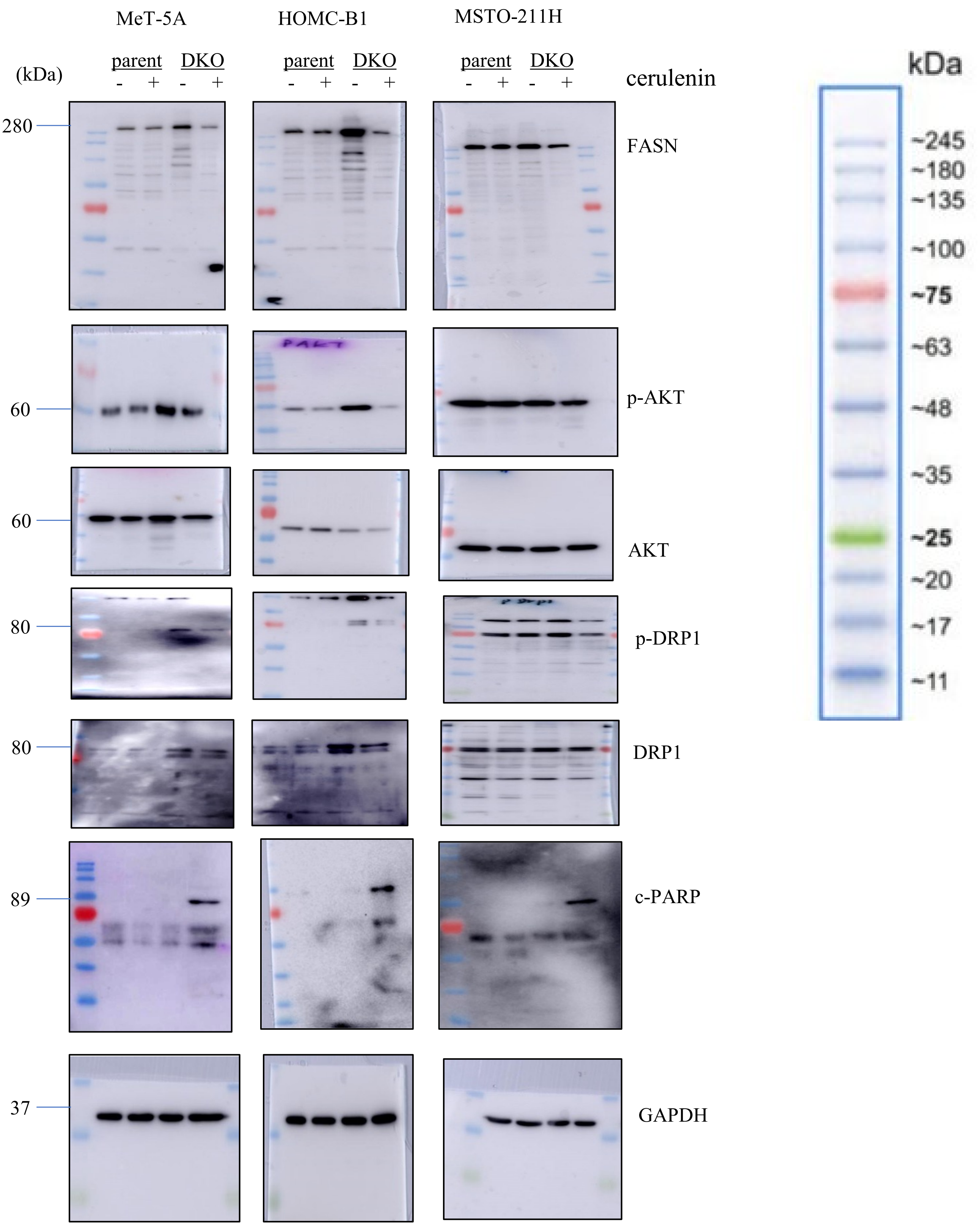


Full and uncropped western blot image of Fig. 4b

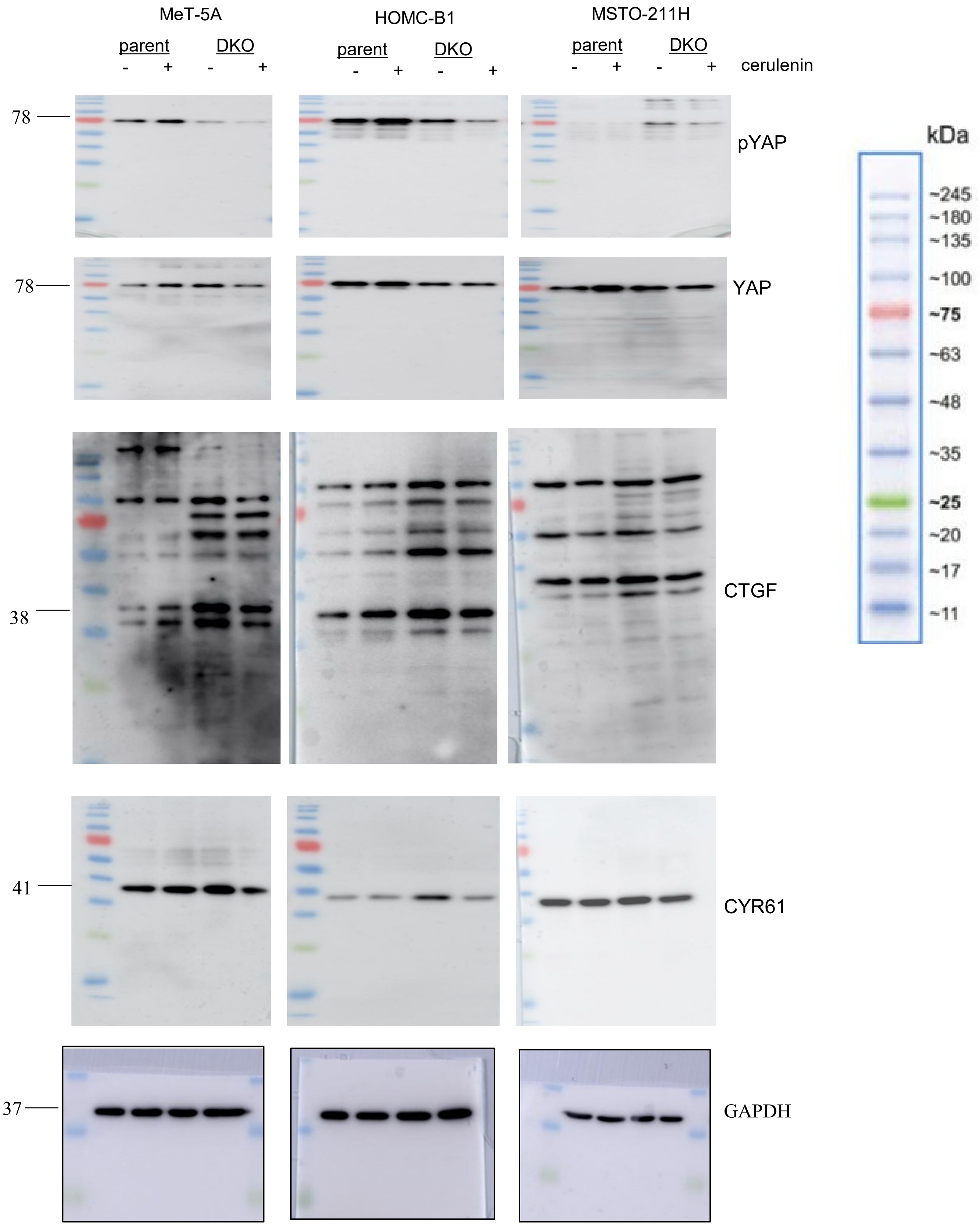


Full and uncropped western blot image of supplementary Fig. 3

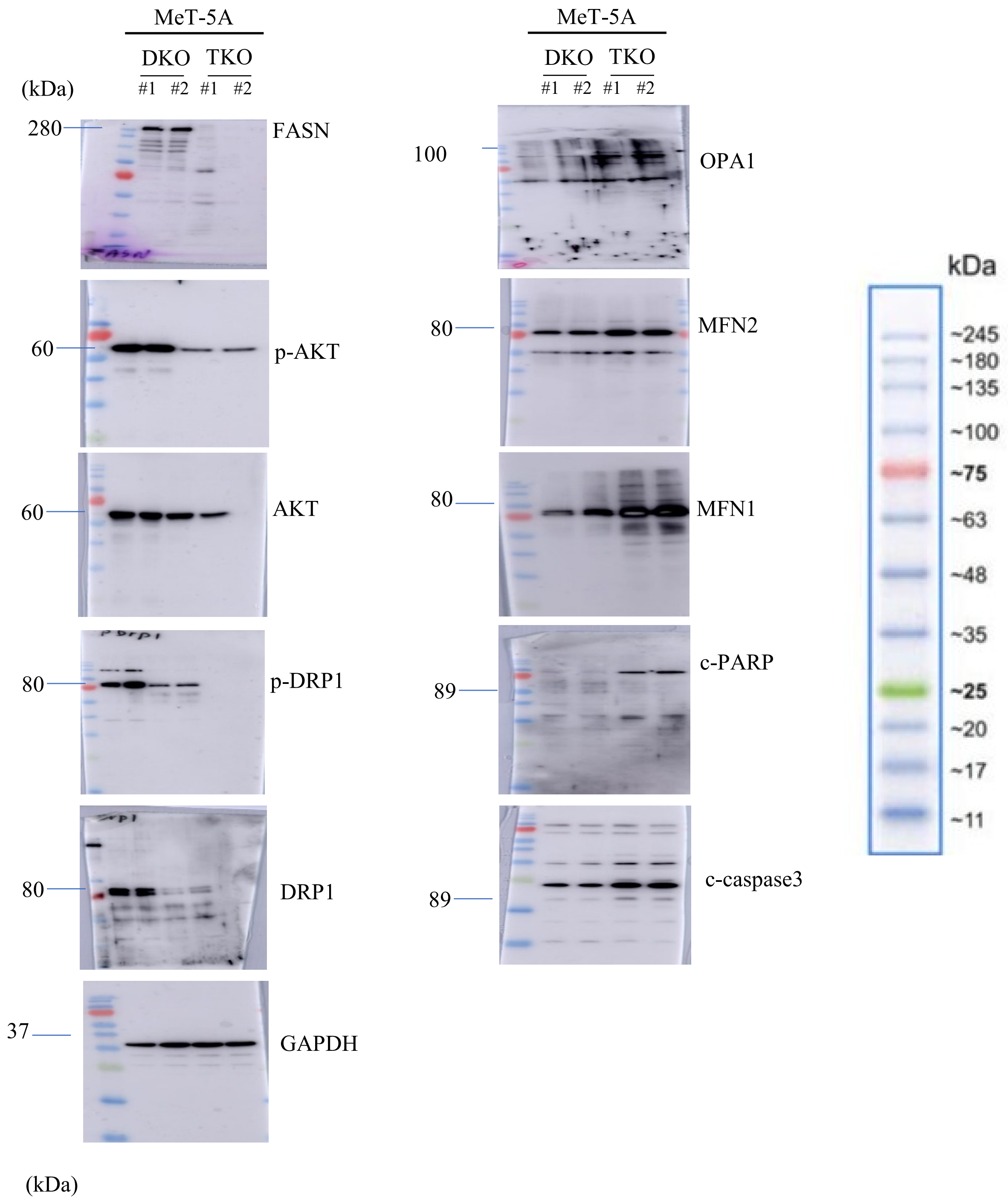


Full and uncropped western blot image of Fig. 5b

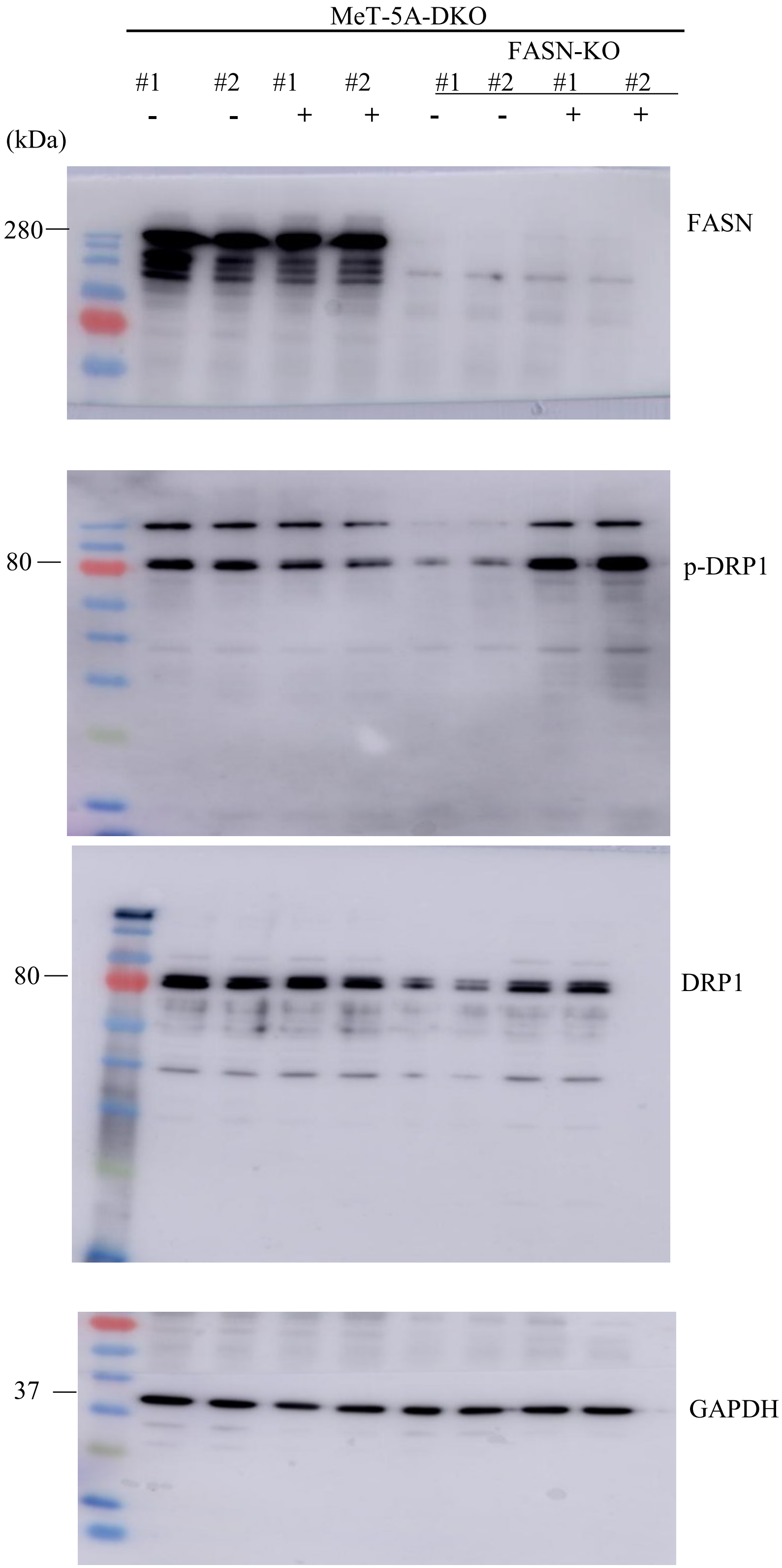


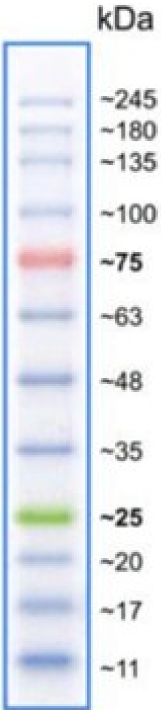


Full and uncropped western blot image of Fig. 5e

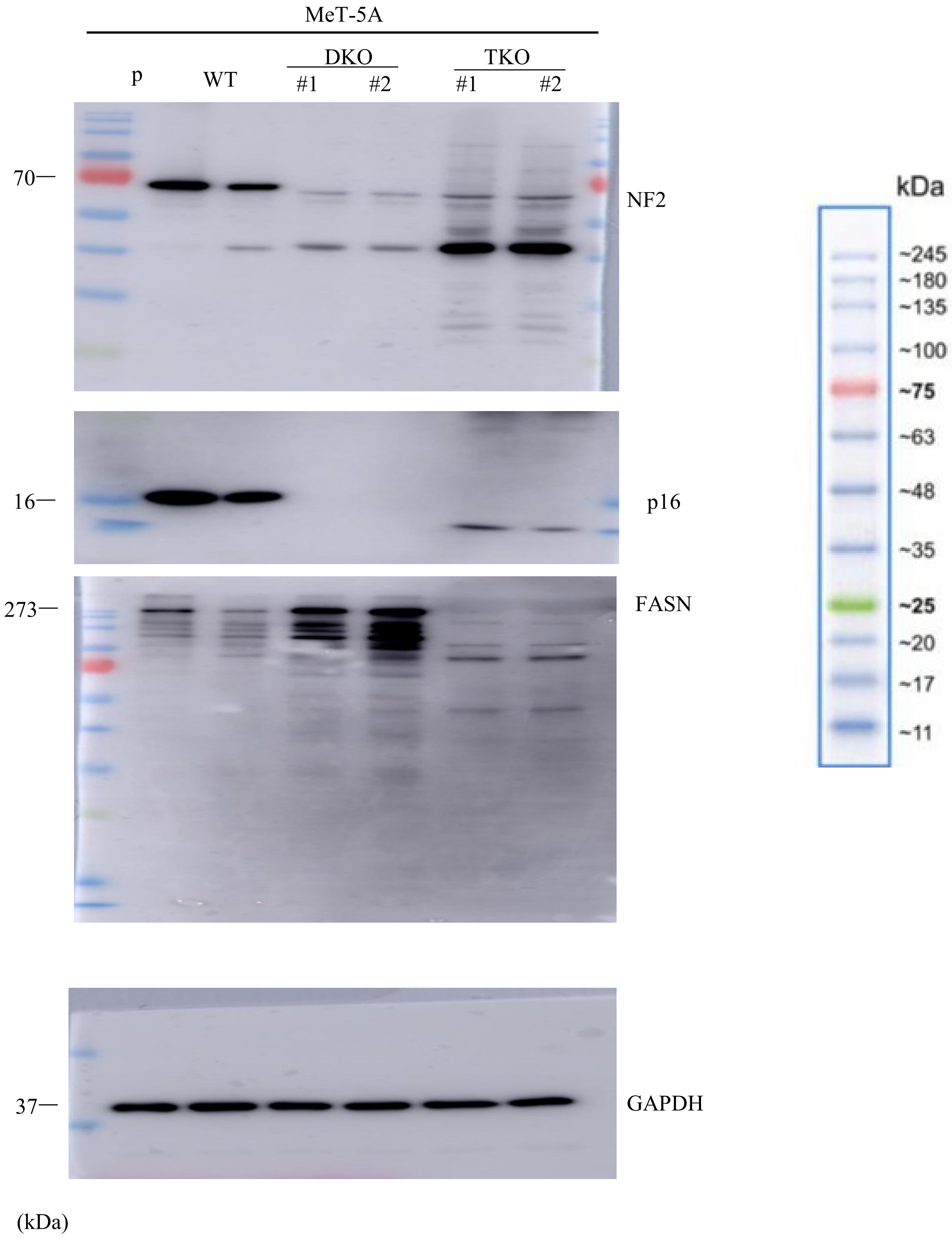


Full and uncropped western blot image of Supplementary Fig. 5c
