# Supplementary material for: Targeting fatty acid synthase suppresses tumor development in NF2/CDKN2A-deficient pleural mesothelioma
Source: Cell Death Dis. 2026 Feb 28;17(1):287. doi: 10.1038/s41419-026-08481-y (PMC13031323; doi:10.1038/s41419-026-08481-y)
Supplement: Supplementary file 3 — Supplementary Table S1 [file 41419_2026_8481_MOESM3_ESM.pdf]

Supplementary Table S1. Effect of 364 compounds on the proliferation of DKO and Parental MeT-5A cells.

No	Category	Compound	MeT-(Parent) cells)	DKO	Relative cell survival
				(%cells)	DKO-parent (%cells)
1	blank	DMSO DMSO	83.88	79.41	-4.47
2	antitumor	5-FU	67.78	67.3	-0.48
3	antitumor	Bestatin	77.45	77.53	0.08
4	antitumor (DNA)	Bleomycin sulfate	22.88	39.67	16.79
5	antitumor (DNA)	Cisplatin	84.6	78.42	-6.18
6	antitumor (DHFR)	Methotrexate	41.39	33.72	-7.67
7	antitumor (DNA)	Mitomycin C	14.35	10.95	-3.4
8	antitumor (tubulin)	Vinblastine sulfate	31.9	36.52	4.62
9	antitumor (tubulin)	Paclitaxel	68.69	47.69	-21
10	antitumor (AR)	Flutamide	81.14	83.62	2.48
11	antitumor (DNA)	Daunorubicin, HCl	15.47	12.98	-2.49
12	antitumor (DNA)	Doxorubicin, HCl	22.11	12.66	-9.45
13	antitumor (ER)	Tamoxifen, citrate	37.94	61.85	23.91
14	antitumor (RNA)	Actinomycin D	20.73	15.59	-5.14
15	antitumor (topo I)	Camptothecin	28.45	31.24	2.79
16	antitumor (topo I/II)	Aclarubicin	19.01	14.48	-4.53
17	antitumor (topo II)	Etoposide (VP-16)	50.27	37.4	-12.87
18	actin filament	Cytochalasin D	88.95	83.63	-5.32
19	adenylcyclase	2',5'-dideoxyadenosine	90.95	81.74	-9.21
20	AKT	AKT inhibitor	85.29	86.19	0.9
21	AKT	NL-71-101	92.79	86.72	-6.07
22	Bcr-Abl	AG957	70.76	72.44	1.68
23	caspase	Z-VAD-FMK	77.32	76.53	-0.79
24	CDC2	Kenpaullone	55.37	59.87	4.5
25	CDK2	Purvalanol A	48.55	49.85	1.3
26	CDK4	3-ATA	60.7	85.05	24.35
27	CDKs	Olomoucine	81.31	80.1	-1.21
28	CKII	TBB	85.41	84.48	-0.93
29	COX-1	Sulindac sulfide	85.16	80.55	-4.61
30	COX-1	Valeryl salicylate	83.25	81.66	-1.59

31	COX-2	NS-398	81.09	81.09	0
32	COX	Sodium salicylate	87.96	82.53	-5.43
33	cyclicphosphodiesterase	Theophylline	83.93	83.6	-0.33
34	DNA methyltransferase	Azacytidine	82.53	84.16	1.63
35	DNA polymerase	Aphidicolin	45.28	64.34	19.06
36	EGFR	AG1478	84.04	82.27	-1.77
37	EGFR, topoII	Genistein	86.32	84.16	-2.16
38	farnesyltransferase	Manumycin A	32.21	56.05	23.84
39	farnesyltransferase	FTI-276	77.9	80.88	2.98
40	Flk-1	SU1498	68.26	76.06	7.8
41	geranylgeranyltransferase I	GGTI-286	64.35	76.09	11.74
42	GR	Dexamethasone	83.75	89.43	5.68
43	GSK-3	GSK-3 inhibitor II	77.37	88.92	11.55
44	HDAC	Scriptaid	23.81	26.38	2.57
45	HDAC	Trichostatin A	24.19	28.82	4.63
46	HER2 (erbB2/neu), EGFR	AG825	82.95	76.63	-6.32
47	protein synthesis	Cycloheximide	34.5	32.39	-2.11
48	HMG-CoA reductase	Lovastatin	39.76	66.47	26.71
49	HSP90	Radicicol	25.72	27.5	1.78
50	HSP90	17-AAG	32.49	86.54	54.05
51	IGF-1R	AG1024	88.42	93.16	4.74
52	iNOS	1400W, HCl	77.91	80.65	2.74
53	iNOS	AMT, HCl	88.87	87.98	-0.89
54	Jak-2	AG490	92.97	83.8	-9.17
55	Jak-2	Cucurbitacin I	22.65	21.21	-1.44
56	JNK	SP600125	71.98	66.96	-5.02
57	lck (p56), TYK	Damnacanthal	46.99	70.43	23.44
58	MEK	PD 98059	82.41	86.45	4.04
59	MEK	U0126	87.63	89.84	2.21
60	methionine aminopeptidase	Fumagillin	68.08	64.61	-3.47
61	MMP	GM 6001	84.19	77.59	-6.6
62	NF-kB	N-Acetyl-L-cysteine	84.49	78.41	-6.08
63	NOS	Aminoguanidine, HCl	85.15	82.45	-2.7

64	NOS	L-NMMA	86.58	82.49	-4.09
65	p38 (MAPK)	PD169316	80.42	72.58	-7.84
66	p38 (MAPK)	SB 203580	75.8	84.48	8.68
67	p70 S6K	Rapamycin	74.45	76.92	2.47
68	PARP	NU1025	82.48	86.49	4.01
69	PARP-1	Benzamide	83.88	81.39	-2.49
70	PC-PLC	D609	85.56	81.69	-3.87
71	PDE	IBMX	83.19	82.39	-0.8
72	PDE (cAMP)	Ro-20-1724	87.14	82.91	-4.23
73	PDE (cGMP)	Zaprinast	84.83	90.26	5.43
74	PDGFR	AG1296	63.99	50.36	-13.63
75	PI3K	LY294002	54.6	67.68	13.08
76	PI3K	Wortmannin	71.82	70.4	-1.42
77	PKA	H-89, HCl	53.55	90.67	37.12
78	PKC	Bisindolymaleimide I, HCl	22.65	30.62	7.97
79	PKC, PKA	H-7	73.07	76.64	3.57
80	PKC, PKA, PKG, MLCK	Staurosporine	13.72	15.43	1.71
81	PLA2	cPLA2inhibitor	89.03	99.16	10.13
82	PLA2	OBAA	61.93	61.6	-0.33
83	PP2A	Cantharidin	84.98	89.81	4.83
84	PP2A	Cytostatin	81.97	90.1	8.13
85	PP2B/cyclophilin	Cyclosporin A	94.14	85.51	-8.63
86	PP2B/FKBP	FK-506	81.08	87.59	6.51
87	proteasome	MG-132	25.6	27.6	2
88	proteasome	Lactacystin	77.62	74.84	-2.78
89	ribonucleotide reductase	Hydroxyurea	88	82.66	-5.34
90	ROCK	HA1077	76.33	78.23	1.9
91	ROCK	Y27632	77.73	83.43	5.7
92	Src, Fyn, Lck	PP1 (analog)	86.13	86.31	0.18
93	Src, Fyn, Lck	PP-H	91.84	90.59	-1.25
94	tubulin depolymerization	Nocodazole	37.61	46.11	8.5
95	tyr phosphatase (PTP)	Dephostatin	89.66	74.29	-15.37
96	p53	Pifithrin-a (cyclic)	87.73	74.52	-13.21

97	p53 activator	PRIMA-1	86.81	80.46	-6.35
98	5α-reductase	Finasteride	89.4	87.09	-2.31
99	aromatase	Aminoglutethimide	87.32	81.82	-5.5
100	aromatase	Formestane	86.74	87.23	0.49
101	progesterone receptor	Mifepristone	86.97	103.54	16.57
102	acetyl-CoA carboxylase	TOFA	86.73	87.33	0.6
103	aminopeptidase A	Amastatin	86.86	75.21	-11.65
104	aminopeptidase M	Actinonin	85.36	77.54	-7.82
105	F1-ATPase	Oligomycin	24.2	48.9	24.7
106	V-ATPase	Bafilomycin A1	13.72	21.06	7.34
107	Bcl-2	HA 14-1	87.19	80.28	-6.91
108	Bcl-XL	BH3I-1	89.22	82.07	-7.15
109	Burton's tyrosine kinase(BTK)	LFM-A13	89.58	88.99	-0.59
110	Burton's tyrosine kinase(BTK)	Terreic acid	81.26	79.63	-1.63
111	calpain	E-64d	88.31	81.5	-6.81
112	calpain, cathepsin B, L	ALLN	96.99	91.77	-5.22
113	cathepsin B	CA-074	90.43	82.64	-7.79
114	cathepsin D	Pepstatin A	92.91	80.65	-12.26
115	cathepsin G	Z-GLF-CMK	25.88	23.89	-1.99
116	CCR2	RS 102895	87.42	77.5	-9.92
117	CCR3	SB 328437	91.23	83.76	-7.47
118	CXCR2	SB 225002	51.98	57.67	5.69
119	CXCR4	AMD3100 octahydrochloride	84.59	76.12	-8.47
120	Cdc25	NSC95397	29.72	28.09	-1.63
121	Cdc25A	SC-αασ9	98.79	87.06	-11.73
122	Na channel	Amiloride	92.16	82.02	-10.14
123	Na channel	Lidocaine	94.54	81.48	-13.06
124	Na ionophore	Monensin	35.85	56.09	20.24
125	Na/K ATPase	Ouabain	30.43	30.66	0.23
126	Na/K/Mg ATPase	Sanguinarine	13.28	9.51	-3.77
127	K channel	Glibenclamide	86.76	77.62	-9.14
128	K channel	Dequalinium	17.37	47.31	29.94
129	K channel opener	Diazoxide	92.66	81.44	-11.22

130	K ionophore	Valinomycin	16.29	38.97	22.68
131	K ionophore	Nigericin	44.98	44.21	-0.77
132	Ca channel	Diltiazem	91.49	76.37	-15.12
133	Ca channel	Nifedipine	90.67	75.4	-15.27
134	Ca channel, MDR	Verapamil	85.26	74.44	-10.82
135	MDR	PGP-4008	50.66	44.43	-6.23
136	BCRP	Fumitremorgin C	94.16	85.51	-8.65
137	Ca ionophore	A23187	13.91	11.05	-2.86
138	Ca ionophore	Ionomycin	17.6	18.29	0.69
139	Ca-ATPase	Thapsigargin	21.58	20.79	-0.79
140	Ca-ATPase	t-Butylhydroquinone (BHQ)	111.61	83.81	-27.8
141	Cl channel	N-phenylanthranilic acid	88.11	81.33	-6.78
142	Cl channel	DIDS	89.05	86.65	-2.4
143	Chk 1	SB 218078	33.28	37.96	4.68
144	Chk 1, 2	Debromohymenialdisine (DBH)	95.44	83.46	-11.98
145	mitochondrial complex I	Rotenone	43.96	44.95	0.99
146	mitochondrial complex III	Antimycin A1	59.97	68.66	8.69
147	CRM1	Leptomycin B*	33.58	38.28	4.7
148	DAG kinase	R59022	97.44	80.66	-16.78
149	DAG kinase	Dioctanoylglycol	92.33	80.58	-11.75
150	DAG lipase	RHC80267	90.58	90.56	-0.02
151	DAG acyltransferase (DGAT)	Xanthohumol	16.18	12.03	-4.15
152	fatty acid synthase (FAS)	C75	88.8	85.6	-3.2
153	FAS	Cerulenin	80.83	32.22	-48.61
154	glycosylation	Tunicamycin	35.88	48.07	12.19
155	glucosidase I, II	Deoxynojirimycin	92.97	79.06	-13.91
156	a-mannosidase	Swainsonine	93.91	81.24	-12.67
157	guanylate cyclase	LY 83583	27.7	25.38	-2.32
158	guanylate cyclase	ODQ	92.94	79.78	-13.16
159	HAT	Anacardic acid	96.63	89.41	-7.22
160	HIF	Chetomin	13.64	10.23	-3.41
161	HIF-1a hydroxylase	Dimethyloxalylglycine	95.11	85.94	-9.17

162	kinesin Eg5	HR22C16	31.11	33.13	2.02
163	kinesin Eg5	Monastrol	94.39	84.4	-9.99
164	lipoxygenase	Nordihydroguaiaretic acid (NDGA)	93.32	84.93	-8.39
165	12, 15-lipoxygenase	ETYA	92.12	81.85	-10.27
166	12-lipoxygenase	Baicalein	63.86	69.08	5.22
167	Mdm2	Nutlin-3	96.99	93.16	-3.83
168	Mdm2	MDM2 inhibitor	99.56	97.13	-2.43
169	monoamine oxidase	Phenelzine	91.64	84.27	-7.37
170	monoamine oxdase B	Deprenyl	93.48	83.01	-10.47
171	mitochondrial permeability transition pore (MPTP)	Decylubiquinone	89.55	77.16	-12.39
172	MPTP	Ro 5-4864	86.69	79.14	-7.55
173	MPTP opener	Lonidamine	92.26	84.66	-7.6
174	myosin light chain kinase	ML-7	79.86	83.86	4
175	O6-methylguanine-DNA methyltransferase (MGMT)	Benzylguanine	88.14	81.92	-6.22
176	ornithine decarboxylase (ODC)	DFMO	92.53	83.78	-8.75
177	PKG	KT 5823	84.31	85.1	0.79
178	PKG	Rp-8-CPT-cGMPS	78.67	75.57	-3.1
179	PPAR-a	MK 886	92.33	90.17	-2.16
180	PPAR-a activator	Clofibrate	91.72	82.5	-9.22
181	PPAR-g	BADGE	91.65	82.09	-9.56
182	PPAR-g activator	Troglitazone	98.44	93.46	-4.98
183	reverse transcriptase	AZT	87.72	86.04	-1.68
184	reverse transcriptase	Nalidixic acid	90.65	84.02	-6.63
185	RNA polymerase	a-Amanitin	38.68	39.9	1.22
186	telomerase	MST-312	46.36	48.58	2.22
187	telomerase	b-Rubromycin	70.35	63.67	-6.68
188	TGF-b receptor	SB 431542	91.74	79.48	-12.26
189	spermidine/spermine N1-acetyltransferase (SSAT) activator	N1,N12-Diethylspermine (BESpm)	85	75.91	-9.09
190	sphingosine N-acyltransferase	Fumonisin B1	83.55	73.19	-10.36
191	AK	ABT-702	63.38	85.31	21.93

192	AKT	Akt Inhibitor IV	10.64	9.23	-1.41
193	AKT	Akt Inhibitor VIII, Isozyme-Selective, Akti-1/2	41.49	75.65	34.16
194	AKT	Akt Inhibitor XI	38.16	74.58	36.42
195	AMPK	compound C	31.62	37.54	5.92
196	ATM	ATM/ATR kinase inhibitor	12.04	34.41	22.37
197	ATM	ATM kinase inhibitor	71.5	79.37	7.87
198	Aurora	Aurora kinase/cdk inhibitor	47.02	45.15	-1.87
199	Aurora	Aurora kinase inhibitor II	105.48	94.36	-11.12
200	Aurora	Aurora kinase inhibitor III	44.22	40.93	-3.29
201	Bcr-abl	AG957	74.7	50.9	-23.8
202	BTK	LFM-A13	94.77	88.91	-5.86
203	BTK	Terreic acid	98.24	79.3	-18.94
204	CAMKII	KN-93	82.43	86.29	3.86
205	CAMKII	KN-62	92.86	107.01	14.15
206	CAMKII	Lavendustin C	104.8	85.3	-19.5
207	CDK	Kenpaullone	70.81	78.26	7.45
208	CDK	purvalanol A	55.13	66.45	11.32
209	CDK	Olomoucine	94.42	81.13	-13.29
210	CDK	Alsterpaullone, 2-cyanoethyl	38.45	41.42	2.97
211	CDK	Cdk1/2 inhibitor III	35.24	33.51	-1.73
212	CDK	Cdk2/9 inhibitor	42.94	42.7	-0.24
213	CDK	NU6102	70.93	71.24	0.31
214	CDK	Cdk4 inhibitor	34.8	47.13	12.33
215	CDK	NSC625987	97.12	81.57	-15.55
216	Chk	SB218078	35.98	43.48	7.5
217	Chk	isogranulatimide	101	83.36	-17.64
218	Chk	Chk2 inhibitor	94.92	78.38	-16.54
219	Chk	Chk2 inhibitor II	89.89	89.96	0.07
220	CK	Ellagic acid	92.71	86.08	-6.63
221	CK	TBB	93.15	88.39	-4.76
222	CK	DMAT	73.02	53.5	-19.52

223	CK	D4476	93.6	75.17	-18.43
224	Clk	TG003	91.82	79.41	-12.41
225	DGK	Diacylglycerol kinase inhibitor II	101.57	88.69	-12.88
226	DNA-PK	IC60211	93.88	82.97	-10.91
227	eEF2	TX-1918	57.34	54	-3.34
228	EGFR	BPIQ-II	90.65	78.17	-12.48
229	EGFR	AG1478	96.4	84.96	-11.44
230	EGFR	AG490	94.42	78.05	-16.37
231	FGFR	SU4984	90.88	75.55	-15.33
232	FGFR	SU5402	78.37	76.22	-2.15
233	Flt-3	Flt-3 Inhibitor	56.82	46.39	-10.43
234	Fms	cFMS Receptor Tyrosine Kinase Inhibitor	95.1	80.51	-14.59
235	Fyn	SU6656	66.99	58.24	-8.75
236	GSK	GSK-3 inhibitor IX	93.93	85.08	-8.85
237	GSK	1-Azakenpaullone	59.77	56.2	-3.57
238	GSK	indirubin-3'-monoxime	59.77	59.79	0.02
239	HER2	AG825	102.58	80.18	-22.4
240	IGF-IR	AG1024	105.15	90.71	-14.44
241	IGF-IR	AGL 2263	106.7	94.08	-12.62
242	IKK	BMS-345541	37.63	45.61	7.98
243	IKK	IKK-2 inhibitor VI	42.36	41.09	-1.27
244	IRAK	IRAK-1/4 inhibitor	95.26	83.23	-12.03
245	Jak	JAK Inhibitor I	105.18	90.11	-15.07
246	Jak	JAK3 Inhibitor VI	104.36	87.24	-17.12
247	JNK	SP600125	91.26	73.77	-17.49
248	JNK	JNK inhibitor VIII	95.02	87.81	-7.21
249	Lck	Damnacanthal	83.65	71.4	-12.25
250	Lck	PP2	66.73	65.77	-0.96
251	MAPK	ERK inhibitor II	85.95	71.61	-14.34
252	MEK	PD98059	96.2	81.87	-14.33
253	MEK	U-0126	99.02	85.82	-13.2
254	MEK	MEK inhibitor I	93.22	83.18	-10.04

255	Met	SU11274	108.06	93.19	-14.87
256	MLCK	ML-7	83.73	90.68	6.95
257	p38	SB202190	86.58	79.73	-6.85
258	p38	SB239063	89.51	73.47	-16.04
259	PDGFR	AG1296	88.79	60.96	-27.83
260	PDGFR	SU11652	11.55	11.92	0.37
261	PDGFR	PDGF receptor tyrosine kinase inhibitor V	103.62	85.62	-18
262	PDGFR	PDGF receptor tyrosine kinase inhibitor IV	51.9	46.3	-5.6
263	PI3K	LY-294002	105.91	90.74	-15.17
264	PI3K	Wortmannin	103.19	91.01	-12.18
265	PKA	H-89	104.39	88.54	-15.85
266	PKA	4-cyano-3-methylisoquinoline	104.92	92.47	-12.45
267	PKC	Bisindolymaleimide I, HCl	32.52	40.55	8.03
268	PKC	Go7874	11.4	9.11	-2.29
269	PKG	Rp-8-CPT-cGMPS	101.8	91.2	-10.6
270	PKG	KT5823	78.59	58.66	-19.93
271	PKR	PKR inhibitor	34.55	43.32	8.77
272	Raf	RAF1 kinase inhibitor I	97.5	87.9	-9.6
273	Raf	ZM 336372	95.74	83.42	-12.32
274	ROCK	H-1152	68.57	70.61	2.04
275	ROCK	Y-27632	90.57	82.91	-7.66
276	Hsp90	radicicol	30.18	33.07	2.89
277	Src	PP1 analog	101.33	91.58	-9.75
278	Syk	Syk inhibitor	62.99	70.18	7.19
279	TGF-βRI	SB431542	96.65	87.58	-9.07
280	TGF-βRI	TGF-b RI kinase inhibitor II	93.76	79.2	-14.56
281	Tpl2	Tpl2 kinase inhibitor	93.4	83.49	-9.91
282	TrkA	TrkA inhibitor	86.07	67.63	-18.44
283	VEGFR	VEGFR receptor tyrosine kinase inhibitor II	74.39	78.41	4.02
284	VEGFR	VEGF recptor 2 kinase inhibitor I	90.78	81.43	-9.35
285	VEGFR	SU1498	93.22	85.17	-8.05

286	Bcr-Abl	nilotinib	96.86	89.27	-7.59
287	Multi-kinases	sorafenib	15.45	32.64	17.19
288	mTOR	temsirolimus	88.41	75.8	-12.61
289	EGFR/Her2	lapatinib	68.01	74.57	6.56
290	Bcr-Abl/Kit	imatinib mesylate	95.2	95.14	-0.06
291	Multi-kinases	sunitinib malate	24.38	33.3	8.92
292	EGFR	gefitinib	94.49	98.76	4.27
293	HDAC	vorinostat	35.69	32.84	-2.85
294	EGFR	erlotinib	93.7	80.7	-13
295	Proteasome	bortezomib	24.04	26.54	2.5
296	Bcr-Abl/Src	dasatinib	42.38	65.16	22.78
297	mTOR	everolimus	72.72	73.62	0.9
298	Multi-kinases	pazopanib	32.89	59.92	27.03
299	Rho/SRF	CCG-1423	90.14	87.65	-2.49
300	PIM	PIM1/2 Kinase Inhibitor V	95.34	91.16	-4.18
301	PIM	PIM1 Inhibitor II	110.86	107.14	-3.72
302	Hedgehog	AY 9944	104.36	100.28	-4.08
303	Hedgehog	cyclopamine	94.36	89.93	-4.43
304	Hedgehog	Jervine	97.14	98.08	0.94
305	STAT3	WP1066	33.25	34.77	1.52
306	STAT3	5,15-DPP	88.79	83.88	-4.91
307	Wnt	IWP-2	102.69	100.72	-1.97
308	Wnt	IWR-1-endo	93.27	97.23	3.96
309	Wnt	FH535	92.25	98.97	6.72
310	Notch	DAPT	98.69	88.69	-10
311	tankyrase-selective PARP	XAV939	90.63	86.91	-3.72
312	pan-PARP	PJ-34	88.49	79.14	-9.35
313	PARP-1/2-selective	Olaparib	61.55	66.34	4.79

314	antipsychotic drug	chlorpromazine hydrochloride	45.16	95.67	50.51
315	depression treatment	desipramine hydrochloride	92.68	94.31	1.63
316	golgi inhibitor	brefeldin A	33.68	36.48	2.8
317	stress inducer	anisomycin	35.27	34.64	-0.63
318	thalidomide family	thalidomide	102.84	92.53	-10.31
319	thalidomide family	lenalidomide	103.66	92.96	-10.7
320	retinoids	tretinoin	96.56	93.77	-2.79
321	retinoids	tamibarotene	96.98	104.8	7.82
322	DNA alkylation	temozolomide	100.07	91.16	-8.91
323	EML4-ALK	crizotinib	13.15	18.46	5.31
324	mTOR	Torkinib	106.02	93.07	-12.95
325	lipase	orlistat	58.79	84.12	25.33
326	AR	MDV3100	101.82	97.42	-4.4
327	caspase activator	PAC-1	39	41.09	2.09
328	blc-2	ABT-737	45.67	42.23	-3.44
329	G9a	UNC0638	20.22	43.22	23
330	G9a	BIX01294	30.08	73.82	43.74
331	LSD1	S2101 (LSD1 inhibitor II)	97.78	90.24	-7.54
332	PRMT1	AMI-1	98.38	89.47	-8.91
333	p300	C646	102.23	93.28	-8.95
334	SIRT1	SIRT1 inhibitor III	79.64	68	-11.64
335	SIRT1/2	Tenovin-6	27.2	58.34	31.14
336	HDAC8	PCI-34051	79.4	69.31	-10.09
337	BRD4 bromodomain	(+)-JQ1	41.9	54.14	12.24
338	Telomerase	TMPyP4	96.09	78.94	-17.15
339	PARP	BSI-201 (Iniparib)	110.67	95.8	-14.87
340	PARP	ABT-888 (Veliparib)	93.94	83.94	-10
341	PARP	AG014699 (Rucaparib)	73.51	72.26	-1.25
342	PARP	MK-4827 (Niraparib)	65.98	55.14	-10.84

343	Aurora	ENMD-2076	106.38	102.53	-3.85
344	Aurora	MLN8237	98.21	85.92	-12.29
345	Survivin	YM155	12.37	9	-3.37
346	PDK1	OSU-03012	88.14	99.61	11.47
347	IGF-IR	OSI-906	68.77	66.67	-2.1
348	c-Met	PF-04217903	91.84	90.92	-0.92
349	DNMT	Decitabine	98.16	87.74	-10.42
350	Multi-kinases	Vandetanib	44.5	62.42	17.92
351	Multi-kinases	Axitinib	62.33	69.44	7.11
352	BRAF	Vemurafenib	67.58	89.01	21.43
353	JAK	Ruxolitinib	65.06	87.18	22.12
354	Hedgehog	Vismodegib	101.13	101.94	0.81
355	GLI1	Gant61	99.4	112.46	13.06
356	FGFR	PD173074	40.32	54.49	14.17
357	ALK	A83-01	91.87	84.43	-7.44
358	GSK-3	BIO	75.72	69	-6.72
359	GSK-3	TWS119	34.86	40.13	5.27
360	GSK-3	CT99021	86.27	106.9	20.63
361	TGFb-R	LY2157299	95.26	88.78	-6.48
362	TGFb-R	SD208	93.07	96.47	3.4
363	ALK	LDN193189	13.36	12.55	-0.81
364	ROCK	Thiazovivin	87.72	80.37	-7.35
