# Supplementary material for: Targeting fatty acid synthase suppresses tumor development in NF2/CDKN2A-deficient pleural mesothelioma
Source: Cell Death Dis. 2026 Feb 28;17(1):287. doi: 10.1038/s41419-026-08481-y (PMC13031323; doi:10.1038/s41419-026-08481-y)
Supplement: Supplementary file 4 — Supplementary Table S2 [file 41419_2026_8481_MOESM4_ESM.pdf]

Supplementary Table S2. Summary of expression status immunohistochemistry in this study

Cases No.	Age	Sex	Pathology diagnosis	subtype	Intensity		
					NF2	p16	FASN
1	64	M	Malignant mesothelioma of chest wall	S	2+	2+	2+
2	48	M	Malignant mesothelioma	S	1+	1+	0
3	58	F	Malignant mesothelioma	B	0	0	2+
4	49	F	Malignant mesothelioma	S	0	0	1+
5	22	M	Malignant mesothelioma	E	0	0	3+
6	49	M	Malignant mesothelioma	E	0	0	1+
7	32	M	Malignant mesothelioma	E	2+	2+	2+
8	29	M	Malignant mesothelioma with necrosis	E	2+	0	3+
9	31	F	Malignant mesothelioma	S	2+	3+	3+
10	70	F	Malignant mesothelioma	E	0	0	2+
11	47	M	Malignant mesothelioma	S	2+	1+	0
12	60	M	Malignant mesothelioma	E	1+	3+	0
13	46	M	Malignant mesothelioma	E	1+	2+	0
14	35	M	Malignant mesothelioma	E	1+	1+	0
15	83	M	Malignant mesothelioma	B	2+	1+	0
16	56	M	Malignant mesothelioma of mediastinal pleura	S	1+	2+	0
17	67	F	Malignant mesothelioma	E	3+	2+	1+
18	40	F	Malignant mesothelioma of left cardiac atrium	S	1+	1+	0
19	50	F	Malignant mesothelioma of left cardiac atrium	E	2+	3+	0
20	43	F	Malignant mesothelioma of pericardium	E	3+	1+	2+
21	43	F	Malignant mesothelioma of pericardium	E	3+	0	2+
22	50	F	Malignant mesothelioma of pericardium	E	2+	0	2+
23	57	M	Malignant mesothelioma	E	0	0	2+
24	29	M	Malignant mesothelioma	E	2+	1+	0

25	71	M	Malignant mesothelioma	E	0	0	2+
26	63	F	Malignant mesothelioma	E	2+	0	2+
27	48	F	Malignant mesothelioma	S	1+	2+	0
28	60	F	Malignant mesothelioma	E	2+	2+	0
29	60	F	Malignant mesothelioma	E	2+	1+	2+
30	33	F	Malignant mesothelioma	E	2+	1+	0
31	47	M	Malignant mesothelioma of mesocolon	E	0	0	2+
32	44	F	Malignant mesothelioma of mesocolon	S	1+	1+	0
33	60	M	Malignant mesothelioma	E	1	3+	0
34	60	M	Malignant mesothelioma	E	0	0	1+
35	76	F	Malignant mesothelioma of hypogastrium	E	2+	3+	2+
36	78	M	Malignant mesothelioma with necrosis of hypogastrium	E	2+	3+	0
37	41	F	Malignant mesothelioma of hypogastrium	S	1+	2+	0
38	28	M	Malignant mesothelioma of retroperitoneum	B	0	0	1+
39	5	F	Malignant mesothelioma of epigastrium	E	0	0	1+
40	56	F	Malignant mesothelioma of mesocolon	E	2+	0	1+
41	45	M	Malignant mesothelioma	E	0	0	1+
42	69	M	Malignant mesothelioma	E	2+	3+	2+
43	78	M	Malignant mesothelioma of hypogastrium	E	0	0	1+
44	33	M	Malignant mesothelioma of retroperitoneum	E	0	0	1+
45	5	F	Malignant mesothelioma	E	0	0	1+
46	15	F	Mesothelium	-	2+	2+	0
47	19	F	Mesothelium	-	2+	1+	0

Epithelioid (E), Sarcomatous (S), Biphasic (B) FASN expression: Epithelioid 23 out of 32, Sarcomatous 3 out of 10, Biphasic 2 out of 3, Each intensity of the positive signal for NF2, 16 and FASN were evaluated by two investigators, scoring staining intensity as strong (3+), moderation (2+), weak (1+), or negative (0).
