# Supplementary material for: Targeting fatty acid synthase suppresses tumor development in NF2/CDKN2A-deficient pleural mesothelioma
Source: Cell Death Dis. 2026 Feb 28;17(1):287. doi: 10.1038/s41419-026-08481-y (PMC13031323; doi:10.1038/s41419-026-08481-y)
Supplement: Supplementary file 5 — Supplementary Table S3 [file 41419_2026_8481_MOESM5_ESM.pdf]

Supplementary Table S3: Blood chemistry in vehicle control mice and Cerulenin-treated mice

Test	Unit	Control<br>(Mean ± SD)	Cerulenin- treated<br>(20mg/kg, 6 times injection)<br>(Mean ± SD)
TP	g/dL	4.20 ± 0.04	4.02 ± 0.05
ALB	g/dL	2.84 ± 0.02	2.78 ± 0.04
BUN	mg/dL	21.62 ± 0.54	21.74 ± 1.28
CRE	mg/dL	0.12 ± 0.01	0.12 ± 0.01
Na	mEq/L	149.80 ± 0.80	152.00 ± 0.75
K	mEq/L	5.30 ± 0.18	4.40 ± 0.13
Cl	mEq/L	110.40 ± 0.40	114.20 ± 0.33
Ca	mg/dL	8.28 ± 0.31	8.02 ± 0.29
IP	mg/dL	12.52 ± 0.97	13.72 ± 0.52
AST	IU/L	48.60 ± 2.66	42.20 ± 1.78
ALT	IU/L	26.60 ± 2.25	23.60 ± 1.12
LDH	IU/L	148.60 ± 13.04	118.40 ± 5.25
AMY	IU/L	2511.20 ± 122.93	2034.60 ± 71.79
γ-GT	IU/L	3＞	3＞
T-CHO	mg/dL	76.40 ± 2.89	73.60± 2.92
TG	mg/dL	93.40 ± 11.98	88.40 ± 3.85
HDL-C	mg/dL	39.00 ± 1.00	39.20 ± 1.71
T-BIL	mg/dL	0.06 ± 0.004	0.04 ± 0.002
GLU	mg/dL	128.60 ± 18.74	177.40 ± 19.00

Abbreviations: TP, total protein; ALB, albumin; BUN, blood urea nitrogen; CRE, creatinine; Na, sodium; K, potassium; Cl, chloride; Ca, calcium; IP, inorganic phosphorus; AST, aspartate aminotransferase; ALT, alanine aminotransferase; LDH, lactate dehydrogenase; AMY, amylase; γ-GT, gamma-glutamyl transferase; T-CHO, total cholesterol; TG, triglycerides; HDL-C, high-density lipoprotein cholesterol; T-BIL, total bilirubin; GLU, glucose (blood sugar); bw, body weight
