# Supplementary material for: Targeting fatty acid synthase suppresses tumor development in NF2/CDKN2A-deficient pleural mesothelioma
Source: Cell Death Dis. 2026 Feb 28;17(1):287. doi: 10.1038/s41419-026-08481-y (PMC13031323; doi:10.1038/s41419-026-08481-y)
Supplement: Supplementary file 6 — Supplementary Table S4 [file 41419_2026_8481_MOESM6_ESM.pdf]

Supplementary Table S4. Antibodies used in this study.

Molecule	Cat number	Company	Species	Dilution
FASN	sc-48357	santa Cruz	Ms	x 2000
Phospho-AKT	#4060	CST	Rb	x 1000
Total-AKT	# 4685	CST	Rb	x 1000
pDRP1	#3455	CST	Rb	x 1000
DRP1	12957-1-AP	Protentech	Rb	x 1000
NF2	sc-332	santa Cruz	Rb	x 2000
p16	ab108349	abcam	Rb	x 2000
MFN1	#14739	CST	Rb	x 2000
MFN2	#9482	CST	Rb	x 2000
Cleaved PARP	#9541	CST	Rb	x 2000
OPA1	sc-393296	santa Cruz	Ms	x 3000
Cleaved -caspase3	#9661	CST	Rb	x 2000
Ubiquitin	sc-8017	santa Cruz	Ms	x 2000
pYAP	#13008	CST	Rb	x 2000
YAP	#14074	CST	Rb	x 2000
CTGF	ab6992	abcam	Rb	x 2000
CYR61	#14479	CST	Rb	x 2000
GAPDH	#2118	CST	Rb	x 2000
Anti-rabbit IgG-HRP	#7074	CST	Goat	x 4000
Hoechst 33342	SW150	Dojindo		x 1000
Alexa Fluor Plus 488	A32723	Invitrogen		x 1000
Alexa Fluor® 568	A11011	Invitrogen		x 1000

CST, Cell Signaling Technology; Rb, rabbit; Ms, mouse
